# VEGF-B prevents excessive angiogenesis by inhibiting FGF2/FGFR1 pathway

**DOI:** 10.1038/s41392-023-01539-9

**Published:** 2023-08-18

**Authors:** Chunsik Lee, Rongyuan Chen, Guangli Sun, Xialin Liu, Xianchai Lin, Chang He, Liying Xing, Lixian Liu, Lasse D. Jensen, Anil Kumar, Harald F. Langer, Xiangrong Ren, Jianing Zhang, Lijuan Huang, Xiangke Yin, JongKyong Kim, Juanhua Zhu, Guanqun Huang, Jiani Li, Weiwei Lu, Wei Chen, Juanxi Liu, Jiaxin Hu, Qihang Sun, Weisi Lu, Lekun Fang, Shasha Wang, Haiqing Kuang, Yihan Zhang, Geng Tian, Jia Mi, Bi-Ang Kang, Masashi Narazaki, Aaron Prodeus, Luc Schoonjans, David M. Ornitz, Jean Gariepy, Guy Eelen, Mieke Dewerchin, Yunlong Yang, Jing-Song Ou, Antonio Mora, Jin Yao, Chen Zhao, Yizhi Liu, Peter Carmeliet, Yihai Cao, Xuri Li

**Affiliations:** 1 State Key Laboratory of Ophthalmology, Zhongshan Ophthalmic Center, Sun Yat-sen University and Guangdong Provincial Key Laboratory of Ophthalmology and Visual Science, Guangzhou, 510060 P. R. China; 2 Affiliated Eye Hospital of Nanjing Medical University, Nanjing, 210000 China; 3 Department of Obstetrics and Gynecology, Guangdong Provincial Key Laboratory of Major Obstetric Diseases,Guangdong Provincial Clinical Research Center for Obstetrics and Gynecology, The Third Affiliated Hospital of Guangzhou Medical University, Guangzhou, China; 4 Shenzhen Eye Hospital, Jinan University, Shenzhen Eye Institute, Shenzhen, China; 5 Department of Health, Medical and Caring Sciences, Division of Diagnostics and Specialist Medicine, Linköping University, 581 83 Linköping, Sweden; 6 Department of Cardiology, Angiology, Haemostaseology and Medical Intensive Care, University Medical Centre Mannheim, Medical Faculty Mannheim, Heidelberg University, Mannheim, Germany; 7 DZHK (German Research Centre for Cardiovascular Research), partner site Mannheim/ Heidelberg, Mannheim, Germany; 8 European Center for Angioscience, Medical Faculty Mannheim, Heidelberg University, Mannheim, Germany; 9 Guangdong Provincial Key Laboratory of Colorectal and Pelvic Floor Disease, Guangdong Research Institute of Gastroenterology, Department of Colorectal Surgery, The Sixth Affiliated Hospital of Sun Yat-sen University, Guangzhou, China; 10 Eye Institute, Eye and ENT Hospital, Shanghai Medical College, Fudan University, Key Laboratory of Myopia of State Health Ministry (Fudan University) and Shanghai Key Laboratory of Visual Impairment and Restoration, 200031 Shanghai, China; 11 Shandong Technology Innovation Center of Molecular Targeting and Intelligent Diagnosis and Treatment, Binzhou Medical University, Yantai, 264003 P. R. China; 12 Division of Cardiac Surgery, National-Guangdong Joint Engineering Laboratory for Diagnosis and Treatment of Vascular Diseases, NHC key Laboratory of Assisted Circulation (Sun Yat-sen University), The First Affiliated Hospital, Sun Yat-sen University, Guangzhou, China; 13 Department of Respiratory Medicine and Clinical Immunology, Graduate School of Medicine, Osaka University, Osaka, 565-0871 Japan; 14 Physical Sciences, Sunnybrook Research Institute, 2075 Bayview Avenue, Toronto, Ontario M4N 3M5 Canada; 15 Laboratory of Angiogenesis and Vascular Metabolism, Department of Oncology, KU Leuven, Leuven, B-3000 Belgium; 16 Laboratory of Angiogenesis and Vascular Metabolism, Center for Cancer Biology (CCB), VIB, Leuven, B-3000 Belgium; 17 Department of Developmental Biology, Washington University School of Medicine, St. Louis, MO 63110 USA; 18 Department of Cellular and Genetic Medicine, School of Basic Medical Sciences, Fudan University, Shanghai, China; 19 Joint School of Life Sciences, Guangzhou Medical University and Guangzhou Institutes of Biomedicine and Health (Chinese Academy of Sciences), Xinzao, Panyu district, Guangzhou, 511436 Guangdong China; 20 Laboratory of Angiogenesis and Vascular Heterogeneity, Department of Biomedicine, Aarhus University, Aarhus, Denmark; 21 Center for Biotechnology, Khalifa University of Science and Technology, Abu Dhabi, United Arab Emirates; 22 Department of Microbiology, Tumor and Cell Biology, Karolinska Institute, 171 77 Stockholm, Sweden

**Keywords:** Cell biology, Molecular biology

## Abstract

Although VEGF-B was discovered as a VEGF-A homolog a long time ago, the angiogenic effect of VEGF-B remains poorly understood with limited and diverse findings from different groups. Notwithstanding, drugs that inhibit VEGF-B together with other VEGF family members are being used to treat patients with various neovascular diseases. It is therefore critical to have a better understanding of the angiogenic effect of VEGF-B and the underlying mechanisms. Using comprehensive in vitro and in vivo methods and models, we reveal here for the first time an unexpected and surprising function of VEGF-B as an endogenous inhibitor of angiogenesis by inhibiting the FGF2/FGFR1 pathway when the latter is abundantly expressed. Mechanistically, we unveil that VEGF-B binds to FGFR1, induces FGFR1/VEGFR1 complex formation, and suppresses FGF2-induced Erk activation, and inhibits FGF2-driven angiogenesis and tumor growth. Our work uncovers a previously unrecognized novel function of VEGF-B in tethering the FGF2/FGFR1 pathway. Given the anti-angiogenic nature of VEGF-B under conditions of high FGF2/FGFR1 levels, caution is warranted when modulating VEGF-B activity to treat neovascular diseases.

## Introduction

VEGF-B was discovered as a VEGF-A homolog in 1996, and is highly expressed in vascular endothelial cells (ECs) and many other cell types.^1,2^ While VEGF-B has been reported to be involved in metabolic complications, such as in diabetes,^3^ the vascular effect of VEGF-B remains poorly understood thus far. Different laboratories, including our own, have reported a pro-angiogenic effect of VEGF-B, mostly under conditions of tissue disintegration or degeneration, such as in myocardial infarction, heart failure or neurodegeneration.^2,4–7^ However, surprisingly and notwithstanding its name, many other studies have reported anti-angiogenic and anti-tumor effects of VEGF-B^8–11^ (Supplementary Table 1). For example, VEGF-B has been shown to inhibit tumor angiogenesis^10^ and tumor growth^9^ in various model systems. Particularly, in cancer patients, high levels of VEGF-B have been shown to be associated with low tumor angiogenesis and better survival,^8,11,12^ and low VEGF-B levels are linked to high tumor angiogenesis and poor patient survival.^8,13^ Importantly, in breast cancer patients, low VEGF-B levels are reported to be related to higher risks of cancer, and high levels lower risks.^11^ Although many studies from different groups have shown anti-angiogenic and anti-tumor effects of VEGF-B, these studies have been largely ignored thus far, with one of the reasons being that the mechanisms involved remain unknown. Yet, despite the poor understanding of the angiogenic effect of VEGF-B, drugs that inhibit VEGF-B together with other VEGF family members are being used in the clinic to treat patients with various neovascular diseases.^14^ However, in many types of cancers, such treatments failed to show benefit.^14,15^ It is therefore urgently imperative to have a better understanding of the angiogenic effect of VEGF-B and the underlying mechanisms.

VEGF-B binds to vascular endothelial growth factor receptor-1 (VEGFR-1),^16^ which also binds to PlGF and VEGF-A. Unlike VEGFR2, VEGFR-1 has a very weak kinase activity, which is about ten-fold weaker than that of VEGFR-2. As such, VEGFR1 can often serve as a decoy receptor to trap certain ligands, such as VEGF-A, to suppress angiogenesis under many conditions.^17^ Noteworthy, in breast cancer patients, high VEGFR1 expression levels have been shown to be associated with better patient survival, while low VEGFR1 levels are linked to adverse cancer phenotypes and poor patient survival.^18,19^ Moreover, in mice, genetic deletion of *Flt1* (encoding VEGFR1) or pharmacological inhibition of VEGFR1 increased adipose angiogenesis.^3^ Furthermore, VEGFR1 has been shown to inhibit Erk activation in vascular ECs,^17,20,21^ which may be at least one of the mechanisms underlying its anti-angiogenic effect. However, it remains thus far unknown whether VEGF-B, as a ligand of VEGFR1, plays a role in the inhibitory effect of VEGFR1.

The fibroblast growth factor 2 (FGF2, also known as basic FGF) is a heparin-binding growth factor expressed in many tissues and cell types, including vascular endothelial cells. FGF2 binds to four receptor tyrosine kinases (FGFR1-4) and acts in a variety of physiological and pathological processes, such as angiogenesis, tumor growth, and development. FGF2 promotes angiogenesis and endothelial cell proliferation and migration.^22–26^ Genetic deletion of *Fgf2* decreases cardiac blood vessel density by about 25%^27^ and impairs angiogenesis.^28–30^ Moreover, FGF2 deficiency results in blood vessel degeneration.^27,28^ FGFR1 is composed of an extracellular region with three immunoglobulin-like domains, a single hydrophobic trans-membrane region, and a cytoplasmic tyrosine kinase domain. FGFR1 is widely expressed, plays important roles in angiogenesis and the phenotype of many types of tumor cells. FGFR1 amplification or upregulation is frequently found in different types of tumors, such as breast cancer, non-small cell lung cancer, ovarian cancer, urothelial carcinoma, and hepatocellular carcinoma. Particularly, in breast cancer, FGFR1 amplification is found in 16–27% of luminal B-type breast cancer patients. Due to its critical role in tumor growth, FGFR1 has been considered as an important drug target for cancer therapy, and multiple small molecule inhibitors against it have been developed. In addition, FGFR1 is essential for the expression and function of VEGF-A and VEGFR2.^29–33^ Despite the abundant expression and potent angiogenic activities of FGF2 and FGFR1, it is thus far not well understood how the potent angiogenic effect of the FGF2/FGFR1 pathway is finely controlled.

In this study, using comprehensive in vitro and in vivo model systems and approaches, including several lines of knockout mice, we unveil an unexpected novel function of VEGF-B as an anti-angiogenic factor to prevent excess angiogenesis by inhibiting the FGF2/FGFR1 pathway when the latter is highly expressed. Mechanistically, we reveal that VEGF-B binds to FGFR1, induces FGFR1/VEGFR1 complex formation, and inhibits FGF2-induced Erk activation. Indeed, in multiple in vitro and in vivo assays, VEGF-B inhibited FGF2-driven angiogenesis and tumor growth. Thus, our work uncovers a previously unknown and surprising function of VEGF-B in restricting the FGF2/FGFR1 pathway. Given the anti-angiogenic nature of VEGF-B under conditions of high FGF2/FGFR1 levels, caution is warranted when modulating VEGF-B activity to treat different types of neovascular diseases.

## Results

### VEGF-B binds to FGFR1

**Fig. 1:**
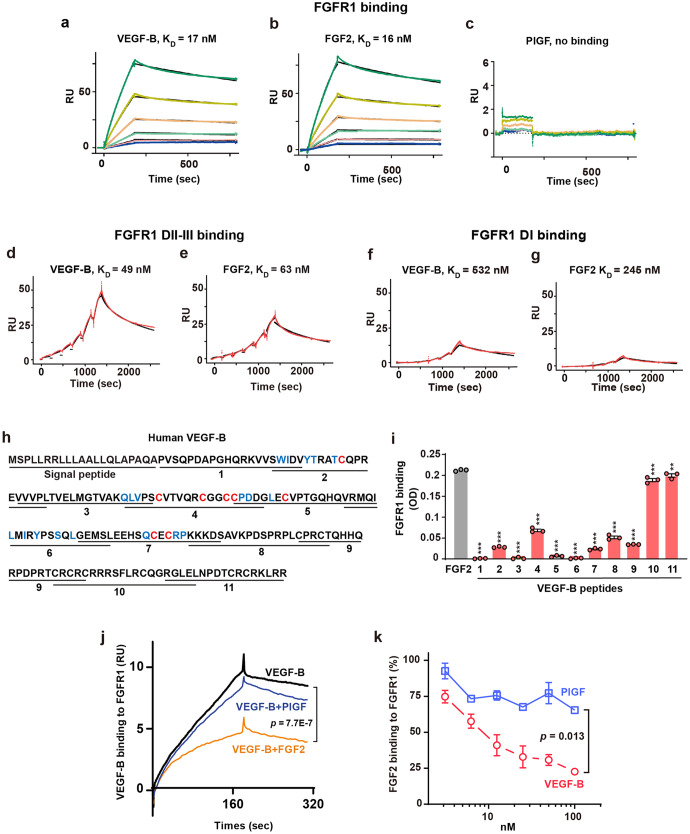
VEGF-B binds to FGFR1 and competes with FGF2 for FGFR1 binding. **a**–**c** Surface plasmon resonance (SPR) results showing VEGF-B binding to FGFR1 with a K_D_ value (17 nM, **a**) similar to that for FGF2 (16 nM, **b**), whereas PlGF, another VEGFR1-binding member of the VEGF family, does not bind to FGFR1 (**c**). The lines from bottom to the top represent different concentrations of FGFR1-Fc: 12.5, 25, 50, 100, 200, and 400 nM, respectively. **d**, **e** SPR results showing VEGF-B binding to FGFR1 DII-III with a K_D_ value (49 nM, **d**) similar to that of FGF2 (63 nM, **e**). The red lines are the resonance unit (RU) values at different concentrations of FGFR1 DII-III (25, 50, 100, 200, 400, 800 nM). The black lines are the fitted curves. **f**, **g** SPR results showing a very low binding affinity of VEGF-B (532 nM, **f**) and FGF2 (245 nM, **g**) to FGFR1 DI. The red lines are the RU values at different concentrations of FGFR1 DI (18.75, 37.5, 75, 150, 300, and 600 nM). The black lines are the fitted curves. **h** Scheme of the synthetic VEGF-B peptides. Blue: amino acids important for VEGFR1 binding. Red: eight cysteines forming the cysteine knots. **i** ELISA results showing the binding of VEGF-B peptides to FGFR1. *n* = 3. One-way ANOVA followed by Sidak post hoc analysis (number of comparisons against FGF2, 11) was used. Adjusted *p* values are <1.0E−10 for peptides 1–9, 1.0E−6 and 5.6E−3 for peptides 10 and 11. The experiment was repeated three times. **j** SPR results showing FGF2 competing with VEGF-B for FGFR1 binding, while PlGF does not. Kruskal-Wallis test with Dunn post hoc analysis (number of comparisons, 3) was used. **k** ELISA results showing VEGF-B dose-dependently competing with FGF2 for FGFR1 binding, while PlGF does not. Data are mean ± s.e.m. The experiment was repeated three times. Two-way ANOVA was used. **p < 0.01, ***p < 0.001

Although many independent studies have reported anti-angiogenic effects of VEGF-B,^8–12^ the mechanisms involved remain unknown. To address this, we screened a phospho-receptor tyrosine kinase (pRTK) antibody array using human retinal endothelial cells (HRECs) to explore in an unbiased manner whether VEGF-B interacts with potentially unknown molecules. Since the VEGF-B_167_ isoform is predominantly expressed in most tissues and accounts for more than 80% of the total VEGF-B transcripts,^34^ we used VEGF-B_167_ in this study and referred to as VEGF-B throughout the whole work. We found that VEGF-B reduced FGFR1 activation (data not shown), which was confirmed by Western blot (Supplementary Fig. 1a, b), suggesting a possible interaction of VEGF-B with FGFR1. We next investigated whether VEGF-B binds to FGFR1. A surface plasmon resonance (SPR) assay showed that VEGF-B binds to FGFR1 with a K_D_ value similar to that of FGF2 (17 nM for VEGF-B and 16 nM for FGF2, Fig. 1a, b), while placental growth factor (PlGF), another VEGFR1-binding member of the VEGF family, showed no binding (Fig. 1c), demonstrating that the binding of VEGF-B to FGFR1 was specific.

### VEGF-B mainly binds to FGFR1 domains II and III

We further investigated which extracellular domains of FGFR1 VEGF-B binds to. For this purpose, we generated recombinant proteins of different extracellular domains (D) of FGFR1: FGFR1 DI, DII, DIII, DI-II, and DII-III, and tested VEGF-B binding using an SPR assay. These recombinant proteins displayed expected molecular weights as shown by both Coomassie blue staining and Western blots (Supplementary Fig. 2a–e). In addition, the functionality of these recombinant proteins was validated by a cell migration assay using human umbilical vein endothelial cells (HUVECs), which express FGFR1 (Supplementary Fig. 2f, g). We found that FGFR1 DII-III reduced FGF2-induced cell migration, while FGFR1 DI-II did not (Supplementary Fig. 2h, i), consistent with previous observations.^35^

An SPR assay showed that VEGF-B bound to FGFR1 DII-III with a K_D_ value similar to that of FGF2 (49 nM for VEGF-B and 63 nM for FGF2; Fig. 1d, e). By contrast, VEGF-B, like FGF2, showed very weak binding to FGFR1 DI (K_D_ values: 532 nM for VEGF-B and 245 nM for FGF2, Fig. 1f, g). Indeed, binding assays using FGFR1 DIII and DII single domains alone further confirmed these observations by showing that VEGF-B bound to FGFR1 DIII and DII with K_D_ values similar to those of FGF2 (FGFR1 DIII: 64 nM for VEGF-B and 43 nM for FGF2; FGFR1 DII: 101 nM for VEGF-B and 71 nM for FGF2, Supplementary Fig. 3a–d). These data all demonstrate that VEGF-B mainly binds to FGFR1 domains II and III.

### Mapping of VEGF-B binding sites for FGFR1

To map the VEGF-B binding sites for FGFR1, we used eleven synthetic VEGF-B peptides covering the entire protein (Fig. 1h, Table 1) and tested whether they bound to FGFR1. An ELISA assay using FGF2 as a positive control revealed that VEGF-B peptides 10 and 11 covering the C terminus of VEGF-B displayed the highest binding activities similar to that of FGF2 (Fig. 1i), with no binding for peptides 1, 3, 5, and 6, and weaker binding for peptides 2, 4, 7, 8, and 9 (Fig. 1i). Noteworthy, the FGFR1-binding VEGF-B peptides 10 and 11 do not overlap with the known amino acid residues important for VEGFR1 binding^36^ (Fig. 1h, blue), suggesting a possibility of simultaneous binding of VEGF-B to FGFR1 and VEGFR1.

**Table 1:** VEGF-B synthetic peptides

Peptides	Sequences
1	PVSQPDAPGHQRKVVSWIDV
2	SWIDVYTRATCQPREVVVPL
3	VVVPLTVELMGTVAKQLVPS
4	QLVPSCVTVQRCGGCCPDDG
5	CPDDGLECVPTGQHQVRMQI
6	VRMQILMIRYPSSQLGEMSL
7	GEMSLEEHSQCECRPKKKDS
8	KKKDSAVKPDSPRPLCPRCT
9	CPRCTQHHQRPDPRTCRCRC
10	CRCRCRRRSFLRCQGRGLEL
11	RGLELNPDTCRCRKLRR

### VEGF-B competes with FGF2 for FGFR1 binding

Since both VEGF-B and FGF2 bind to FGFR1, we used complementary methods to explore whether they compete with each other for FGFR1 binding. First, an SPR analysis demonstrated that FGF2 competed with VEGF-B for FGFR1 binding, while PlGF did not (Fig. 1j). Second, a competitive ELISA showed that VEGF-B, but not PlGF, dose-dependently competed with FGF2 for FGFR1 binding (Fig. 1k). These results thus confirmed each other and suggested a possibility that VEGF-B might inhibit FGF2’s function by competing for FGFR1 binding.

### VEGF-B induces VEGFR1/FGFR1 complex formation

**Fig. 2:**
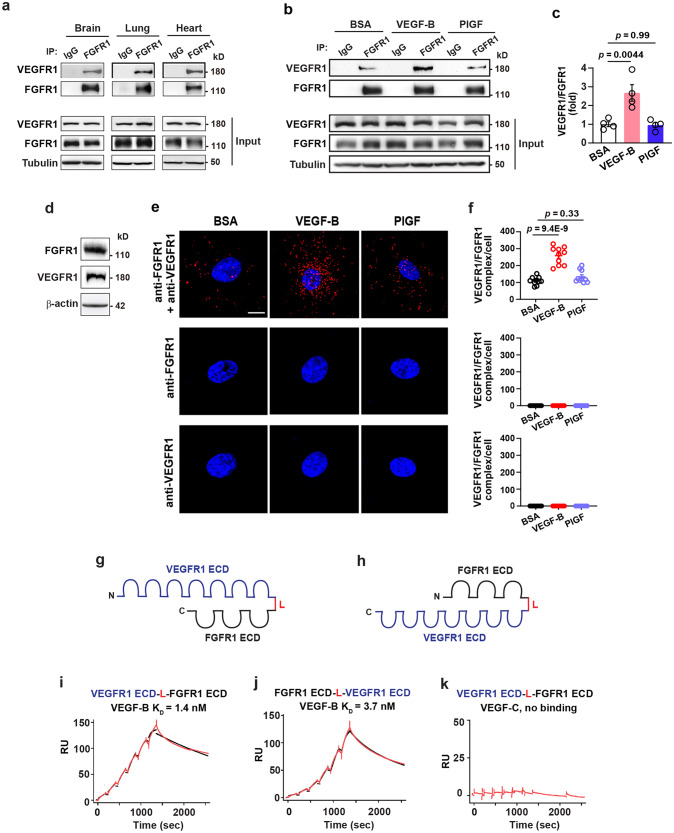
VEGF-B induces VEGFR1/FGFR1 complex formation and binds to VEGFR1/FGFR1 heterodimer with a high affinity. **a** Immunoprecipitation (IP) followed by Western blot showing VEGFR1 co-precipitated with FGFR1 in mouse brain, lung and heart. **b**, **c** Immunoprecipitation followed by Western blot showing that VEGF-B, but not PlGF, induced VEGFR1/FGFR1 complex formation in mouse retinae. **d** Western blot showing the expression of VEGFR1 and FGFR1 in HRECs. **e**, **f** Representative images (**e**) and corresponding quantification (**f**, *n* = 10 per group. The experiment was repeated three times) of in situ proximity ligation assays showing that 30 min treatment of VEGF-B (50 ng/ml), but not PlGF (50 ng/ml), induced VEGFR1/FGFR1 complex formation in HRECs (top panel). Adding one antibody alone at a time did not induce any complex formation (anti-FGFR1: middle panel; anti-VEGFR1: lower panel). Blue: DAPI; red: VEGFR1/FGFR1 complex. Data are mean ± s.e.m., *n* = 4 for (**c**) and 10 for (**f**). For (**c**) and (**f**), adjusted *p* values are from one-way ANOVA followed by Sidak post hoc analysis (number of comparisons, 2). Scale bar: 10 µm. The experiment was repeated three times. **g**, **h** Schemes of recombinant VEGFR1/FGFR1 (**g**) and FGFR1/VEGFR1 (**h**) heterodimers, each containing the extracellular domain (ECD) of VEGFR1 and FGFR1 connected by a linker (L). **i**–**k** SPR results showing VEGF-B binding to the VEGFR1/FGFR1 heterodimer (**i**) and FGFR1/VEGFR1 heterodimer (**j**) with similar K_D_ values, while VEGF-C shows no binding (**k**). The red lines are the RU values at different concentrations of VEGFR1/FGFR1 heterodimers (1.875, 3.75, 7.5, 15, 30, and 60 nM). The black lines are the fitted curves

Given that VEGF-B binds to VEGFR1^16^ and FGFR1 (Fig. 1a, d, i), we explored whether VEGF-B could induce VEGFR1/FGFR1 complex formation. Immunoprecipitation (IP) followed by Western blot showed that VEGFR1 co-immunoprecipitated with FGFR1 in mouse tissues, such as brain, lung, and heart (Fig. 2a, Supplementary Fig. 4a), demonstrating the existence of a naturally occurring endogenous VEGFR1/FGFR1 complex. Importantly, intravitreal injection of VEGF-B into mouse eyes increased the amount of VEGFR1/FGFR1 complex in the retinae, while PlGF did not (Fig. 2b, c, Supplementary Fig. 4a). To directly visualize the FGFR1/VEGFR1 complex, we performed an in situ proximity ligation assay using HRECs expressing both VEGFR1 and FGFR1 (Fig. 2d, Supplementary Fig. 4a), and found that VEGF-B treatment increased the association of FGFR1 with VEGFR1, while PlGF did not (Fig. 2e, f, upper panels), demonstrating that the effect of VEGF-B was specific. FGFR1 and VEGFR1 levels were not changed after the treatment of VEGF-B or PlGF in the HRECs (Supplementary Fig. 4b, c). Adding one antibody alone at a time did not induce any complex formation (Fig. 2e, f, middle and lower panels). In addition, in situ proximity ligation assay also revealed that intravitreal injection of VEGF-B into mouse eyes increased VEGFR1/FGFR1 complex formation in mouse retinae, while PlGF did not (red dots, Supplementary Fig. 5a, b). Thus, various in vivo and in vitro assays show that VEGF-B induces FGFR1/VEGFR1 complex formation.

### VEGF-B binds to VEGFR1/FGFR1 heterodimer with a high affinity

Since VEGF-B induces VEGFR1/FGFR1 complex formation, we subsequently explored whether VEGF-B binds to the VEGFR1/FGFR1 heterodimer. To address this, we generated soluble VEGFR1/FGFR1 heterodimer recombinant proteins containing the extracellular domains (ECDs) of VEGFR1 and FGFR1 connected by a linker (L) and conjugated with a histidine tag (His, Fig. 2g, h). Also, VEGFR1 and FGFR1 ECDs were arranged in different orders, thus resulting in two types of recombinant heterodimers: VEGFR1 ECD-L-FGFR1 ECD and FGFR1 ECD-L-VEGFR1 ECD (Fig. 2g, h). These recombinant proteins displayed expected molecular weights as shown by Coomassie blue staining and Western blot and did not form aggregates (Supplementary Fig. 6a, b). SPR analysis showed that VEGF-B bound to each heterodimer with similar K_D_ values, while VEGF-C showed no binding (Fig. 2i–k), demonstrating that the binding of VEGF-B was specific. The K_D_ values of VEGF-B for VEGFR1 ECD-L-FGFR1 ECD and FGFR1 ECD-L-VEGFR1 ECD were 1.4 nM and 3.7 nM, respectively (Fig. 2i, j). Noteworthy, and importantly, the binding affinities of VEGF-B for the VEGFR1/FGFR1 heterodimers (1.4–3.7 nM) are higher than those for the FGFR1 homodimer (17 nM, Fig. 1a), suggesting that VEGF-B may preferably bind to the VEGFR1/FGFR1 heterodimer.

### VEGF-B inhibits FGF2-induced Erk activation

**Fig. 3:**
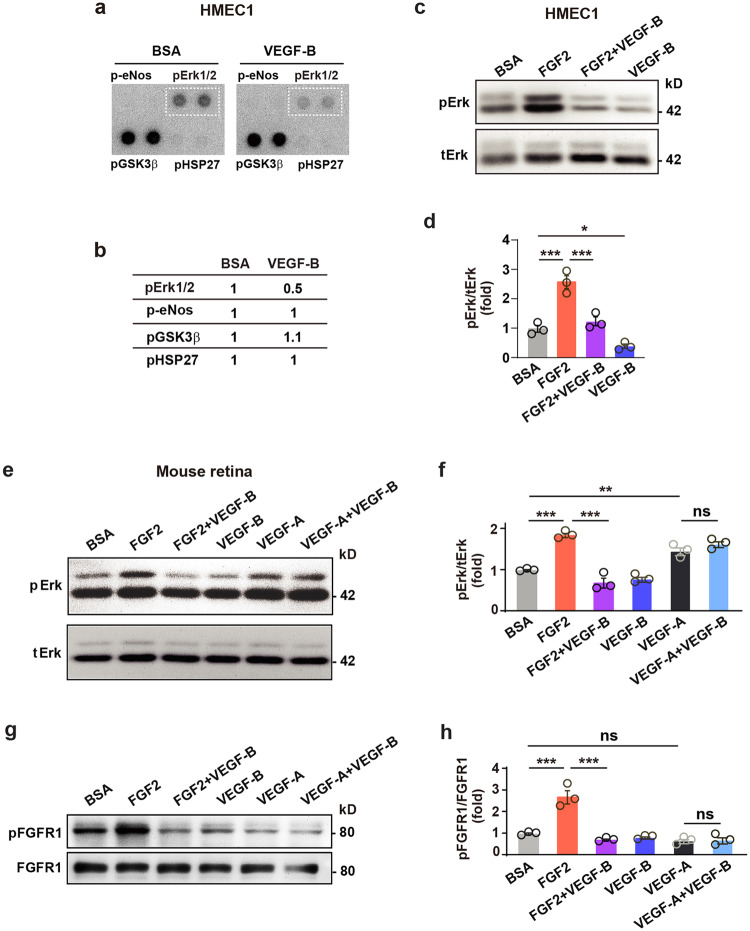
VEGF-B inhibits FGF2-induced Erk activation in vitro and in vivo. **a**, **b** Images of phospho-kinase antibody array screening (**a**) using HMEC1s and corresponding quantifications (**b**). **c**, **d** Western blots showing that VEGF-B (100 ng/ml, 10 min treatment) reduced FGF2 (50 ng/ml)-induced Erk phosphorylation in HMEC1. One-way ANOVA followed by Holm-Sidak post hoc analysis was used (number of comparisons, 3). Adjusted *p* values are 2.1E−4 for BSA vs FGF2; 4.0E−4 for FGF2 vs FGF2 + VEGF-B, and 0.021 for VEGF-B vs BSA. **e**, **f** Western blots showing that in mouse retinae, intravitreal injection of VEGF-B inhibited FGF2-, but not VEGF-A-induced Erk phosphorylation (30 min after injection). Adjusted *p* values are 1.1E−5 for BSA vs FGF2; 1.5E−7 for FGF2 vs FGF2 + VEGF-B; 2.7E−3 for VEGF-A vs BSA and 0.14 for VEGF-A vs VEGF-A + VEGF-B. **g**, **h** Western blots showing that in mouse retinae, intravitreal injection of VEGF-B inhibited FGF2-induced FGFR1 phosphorylation (30 min after injection). Adjusted *p* values are 8.2E−6 for BSA vs FGF2; 5.5E−7 for FGF2 vs FGF2 + VEGF-B and 0.85 for VEGF-A vs VEGF-A + VEGF-B. For (**f**) and (**h**), two-way ANOVA followed by Sidak post hoc analysis was used (number of comparisons, 9). *n* = 3 each group. *p < 0.05, **p < 0.01, ***p < 0.001, ns: *p* > 0.05. The experiments were repeated three times

To investigate VEGF-B-induced downstream signals, we screened a phospho-kinase antibody array using different types of ECs, and found that VEGF-B decreased Erk phosphorylation in human dermal microvascular endothelial cells 1 (HMEC1, Fig. 3a, b, Supplementary Fig. 7), HRECs and HUVECs (Supplementary Fig. 8a, b), while not affecting other molecules, such as eNOS, GSK3ß, and HSP27 (Fig. 3a, b, Supplementary Fig. 9a, b). FGF2 is abundantly expressed in HUVECs, HMEC1s, and HRECs (Supplementary Fig. 9c). Indeed, these findings were confirmed by Western blots in HMEC1 cells (Fig. 3c, d, Supplementary Fig. 7). Importantly, in mouse retinae in vivo, intravitreal injection of VEGF-B inhibited FGF2-, but not VEGF-A-induced Erk phosphorylation (Fig. 3e, f, Supplementary Fig. 7), demonstrating that the inhibitory effect of VEGF-B was specific. Noteworthy, the inhibitory effect of VEGF-B on FGF2-induced FGFR1 activation was confirmed in vivo in mouse retinae (Fig. 3g, h, Supplementary Fig. 7). We further tested whether neuropilin 1 (NRP1) or heparin affected the inhibitory effect of VEGF-B on FGF2-induced Erk phosphorylation. We found that NRP1 knockdown (Supplementary Fig. 10a, b and Supplementary Fig. 11) or administration of heparin (Supplementary Fig. 10c, d and Supplementary Fig. 11) did not affect the inhibitory effect of VEGF-B on FGF2-induced Erk phosphorylation in HRECs.

**Fig. 4:**
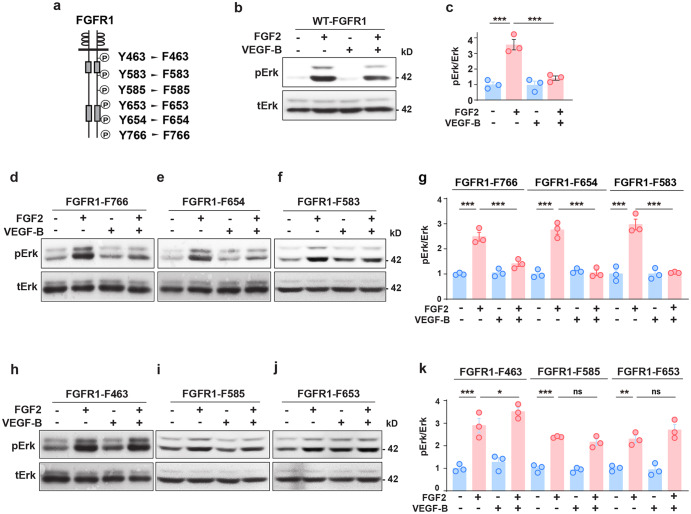
FGFR1 tyrosine residues important for the inhibitory effect of VEGF-B on FGF2-induced Erk activation. **a** Scheme showing the tyrosine (Y) residues of FGFR1 replaced by phenylalanine (F). **b**–**g** Western blots showing that VEGF-B (100 ng/ml, 30 min treatment) inhibits FGF2 (50 ng/ml)-induced Erk phosphorylation in cells expressing wild-type FGFR1 (WT-FGFR1, **b**, **c**) or the FGFR1-F766 (**d**, **g**), FGFR1-F654 (**e**, **g**), FGFR1-F583 (**f**, **g**) mutants. *n* = 3 each group. For (**c**), adjusted *p* values are 9.7E−5 for FGF2 vs. BSA and 3.4E−4 for FGF2 vs FGF2 + VEGF-B. For FGFR1-F766, FGFR1-F654, and FGFR1-F583 in (**g**), adjusted *p* values are 4.0E−8 for BSA vs FGF2; 7.4E−6 for FGF2 vs FGF2 + VEGF-B; 1.2E−5 for FGF2 vs. BSA; 1.4E−5 for FGF2 vs FGF2 + VEGF-B; 2.9E−5 for FGF2 vs. BSA and 3.6E−5 for FGF2 vs FGF2 + VEGF-B. **h**–**k** Western blots showing that in cells expressing the FGFR1-F463 (**h**, **k**), FGFR1-F585 (**i**, **k**), or FGFR1-F653 (**j**, **k**) mutants, VEGF-B failed to inhibit FGF2-induced Erk phosphorylation. Adjusted *p* values are 6.0E−8 for FGFR1-F463; 1.2E−5 for FGFR1-F585 and 1.3E−3 for FGFR1-F653. For (**c**), (**g**), and (**k**), one-way ANOVA followed by Sidak post hoc analysis was used (number of comparisons is 2 for **c**, **g**, and **k**). All data are mean ± s.e.m. *p < 0.05, **p < 0.01, ***p < 0.001, ns: *p* > 0.05. The experiments were repeated three times

We further identified the tyrosine residues in the cytoplasmic part of FGFR1 important for the inhibitory effect of VEGF-B on FGF2-induced Erk activation. We generated a series of site-directed mutations of six tyrosines (Y) of FGFR1 by replacing each of them with a phenylalanine (F), respectively (Fig. 4a, Y463 to F463; Y583 to F583; Y585 to F585; Y653 to F653; Y654 to F654; Y766 to F766), and expressed each mutant in HeLa cells, which express little endogenous FGFR1.^37^ While VEGF-B inhibited FGF2-induced Erk phosphorylation in cells expressing wild-type FGFR1 (WT-FGFR1, Fig. 4b, c) and the FGFR1-F766, FGFR1-F654, and FGFR1-F583 mutants (Fig. 4d–g), it failed to do so in cells expressing the FGFR1-F463, FGFR1-F585, and FGFR1-F653 mutants (Fig. 4h–k), suggesting that tyrosine residues Y463, Y585, and Y653 are important for the inhibitory effect of VEGF-B. We further performed immunoprecipitation assays and investigated whether tyrosine residues Y463, Y585, or Y653 of FGFR1 mediated VEGF-B-induced VEGFR1/FGFR1 complex formation. We found that VEGF-B increased VEGFR1/FGFR1 complex formation in HUVECs overexpressing wild-type FGFR1 (FGFR1 WT), but not in the HUVECs overexpressing the mutants of FGFR1 Y463F, FGFR1 Y585F, or FGFR1 Y653F (Supplementary Figs. 12a, b, 13), suggesting roles of tyrosine residues Y463, Y585, and Y653 in mediating VEGF-B-induced VEGFR1/FGFR1 complex formation. Thus, both in vitro and in vivo data demonstrated an inhibitory effect of VEGF-B on FGF2-induced Erk activation.

### VEGF-B inhibits FGF2-driven angiogenesis and tumor growth

**Fig. 5:**
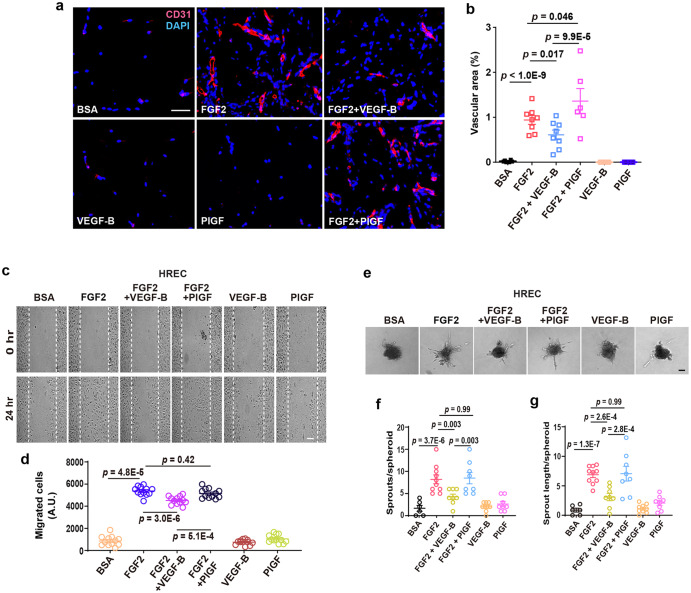
VEGF-B inhibits FGF2-induced angiogenesis. **a**, **b** Representative images (**a**) and corresponding quantification (**b**) of in vivo Matrigel assay showing that VEGF-B inhibits FGF2-induced angiogenesis. *n* = 8 for FGF2 and FGF2 + VEGF-B, *n* = 6 for BSA, VEGF-B, FGF2 + PlGF and PlGF. *p* values are from two-way ANOVA followed by LSD test using the logarithmically transformed data. **c**, **d** Representative images (**c**) of cell migration assays using HRECs and corresponding quantifications of migrated cells (**d**, *n* = 12 per group). **e**–**g** Representative images (**e**) of HREC spheroid sprouting assays and corresponding quantifications of the number of sprouts/spheroid (**f**) and sprout length (**g**). *n* = 6, 9, and 10 per group for BSA, VEGF-B (100 ng/ml) and FGF2 (50 ng/ml); *n* = 8 per group for FGF2 (50 ng/ml) + VEGF-B (100 ng/ml), FGF2 (50 ng/ml) + PlGF (100 ng/ml) and PlGF (100 ng/ml). The experiment was repeated three times. For (**d**), (**f**), and (**g**), adjusted *p* values are from two-way ANOVA followed by Sidak post hoc analysis (number of comparisons, 9). Scale bars: 50 µm for (**a**) and (**c**), 100 µm for (**e**). A.U.: arbitrary unit. All data are mean ± s.e.m. The experiments were repeated three times

Since VEGF-B inhibits FGF2-induced Erk phosphorylation, we subsequently explored whether VEGF-B affected the angiogenic activity of FGF2 using multiple assays. An in vivo subcutaneous Matrigel assay showed that VEGF-B inhibited FGF2-induced angiogenesis, while PlGF showed no effect, demonstrating that the inhibitory effect of VEGF-B was specific (Fig. 5a, b). Moreover, a monolayer cell migration assay revealed that VEGF-B, but not PlGF, suppressed FGF2-induced migration in both HRECs (Fig. 5c, d) and HUVECs (Supplementary Fig. 14a, b). Furthermore, an EC spheroid assay showed that VEGF-B inhibited FGF2-induced EC sprouting in both HRECs (Fig. 5e–g) and HUVECs (Supplementary Fig. 14c–e), while PlGF had no effect, demonstrating that the inhibitory effect of VEGF-B was specific.

**Fig. 6:**
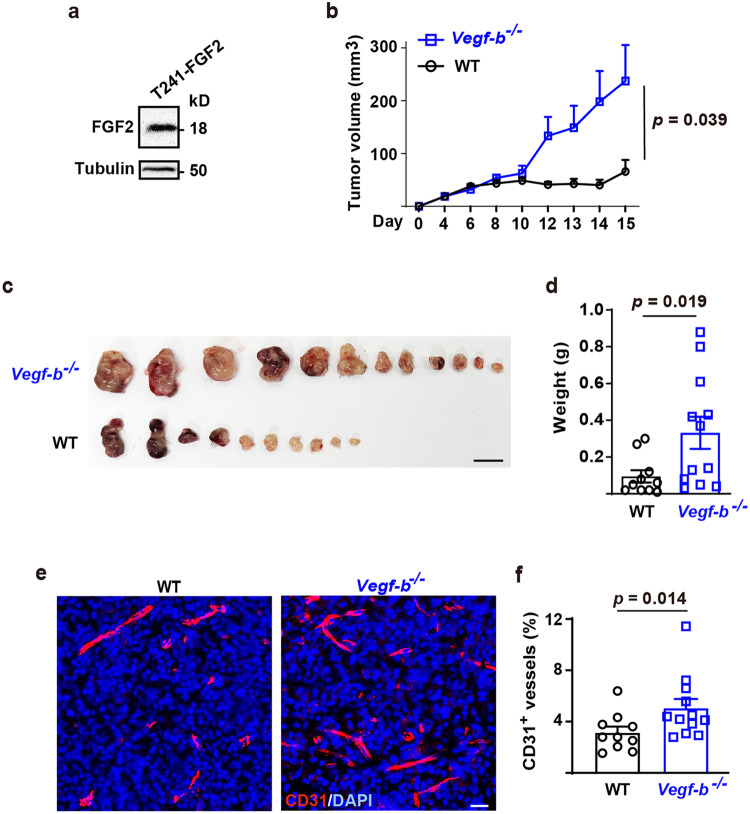
VEGF-B inhibits FGF2-overexpressing tumor growth and tumor angiogenesis. **a** Western blot showing the abundant expression of FGF2 in murine T241 fibrosarcoma cells (T241-FGF2). **b** Tumor growth curves showing that the T241-FGF2 cells formed bigger tumors in *Vegf-b*^−/−^ mice than in wild-type littermates. *P* value was from two-way ANOVA followed by LSD multiple comparisons test. **c** Images showing that the T241-FGF2 cells formed bigger tumors in *Vegf-b*^−/−^ than in wild-type mice. Scale bar: 1 cm. **d** Shown are weights of the tumors from (**c**). *P* value was from Mann-Whitney test. **e** Representative images showing that T241-FGF2 tumor angiogenesis was higher in *Vegf-b*^−/−^ mice than in wild-type littermates. Scale bar: 20 µm. **f** Quantifications of tumor blood vessel densities in (**c**). *P* value was from Mann-Whitney test. All data are mean ± s.e.m. The experiment was repeated twice

We further verified the in vivo relevance of the anti-angiogenic effect of VEGF-B using murine T241 fibrosarcoma cells expressing a high level of FGF2 (T241-FGF2, Fig. 6a) and investigated tumor angiogenesis and tumor growth in *Vegf-b*^−/−^ mice (produced by the Jackson Laboratory, https://www.komp.org/, Supplementary Fig. 15a, b). We found that the T241-FGF2 cells formed significantly bigger tumors in *Vegf-b*^−/−^ mice (Fig. 6b–d) with increased tumor angiogenesis (Fig. 6e, f), even though the T241-FGF2 cells expressed VEGF-B (Supplementary Fig. 15c, d), which might partially compensated for the loss of VEGF-B in *Vegf-b* deficient mice. Together, multiple in vitro and in vivo assays demonstrated an inhibitory effect of VEGF-B on FGF2-mediated functions.

### VEGFR1 and FGFR1 are required for the inhibitory effect of VEGF-B

**Fig. 7:**
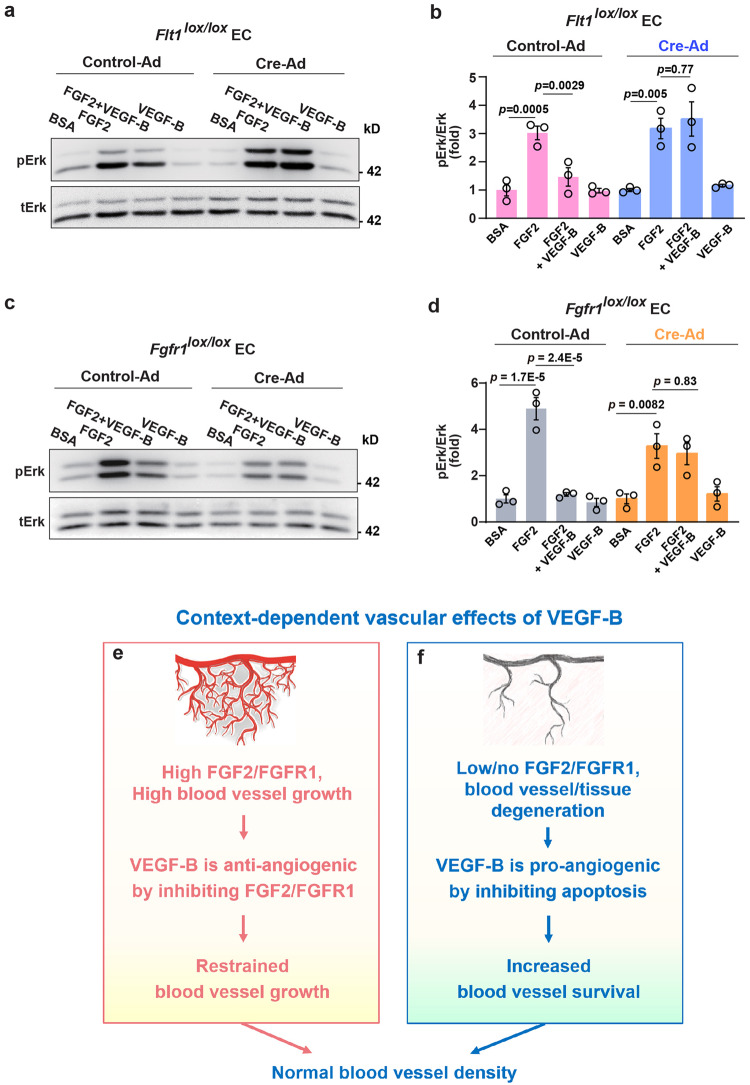
VEGFR1 and FGFR1 are required for the inhibitory effect of VEGF-B. **a**, **b** Western blot showing that VEGF-B (10 min treatment) inhibits FGF2-induced Erk activation in WT ECs (Control-Ad). This inhibition is lost upon *Flt1* deletion (Cre-Ad). **c**, **d** Western blot showing that VEGF-B (100 ng/ml, 10 min treatment) inhibits FGF2 (50 ng/ml)-induced Erk activation in WT ECs (Control-Ad). This inhibition is lost upon *Fgfr1* deletion (Cre-Ad). For (**b**) and (**d**), adjusted *p* values were from one-way ANOVA followed by Sidak post hoc analysis (number of comparisons, 2). The experiments were repeated three times. **e**, **f** Scheme illustrating the context-dependent effects of VEGF-B. Under conditions of high FGF2/FGFR1 levels, VEGF-B can be anti-angiogenic by inhibiting the FGF2/FGFR1 pathway, such as in tumors characterized by abundant FGF2/FGFR1 expression (**e**). Under conditions of low/no FGF2/FGFR1 expression, such as in tissue/blood vessel disintegration (e.g., myocardial infarction or blood vessel regression after FGF2 withdrawal), VEGF-B can be pro-angiogenic due to its known anti-apoptotic and survival effects (**f**). Therefore, depending on FGFR1/FGF2 levels, VEGF-B can be anti- or pro-angiogenic depending on the specific condition

Since VEGF-B binds to VEGFR1 (Flt1) and FGFR1, we examined whether they played a role in the inhibitory effect of VEGF-B using primary lung vascular ECs from *Flt1*^lox/lox^ and *Fgfr1*^lox/lox^ mice with *Flt1* or *Fgfr1* deleted, respectively, using Cre adenovirus (Cre-Ad) as confirmed by Western blots (Supplementary Fig. 16a, b). We found that while VEGF-B inhibited FGF2-induced Erk activation in wild-type ECs (Control-Ad, Fig. 7a–d), this inhibitory effect diminished after *Flt1* or *Fgfr1* deletion, respectively, by Cre-Ad (Fig. 7a–d).

## Discussion

In this study, we report our novel finding that VEGF-B, a member of the VEGF family, under conditions of high FGF2/FGFR1 levels, functions as an anti-angiogenic factor by suppressing the FGF2/FGFR1 pathway. Mechanistically, we discovered that VEGF-B binds to FGFR1, induces FGFR1/VEGFR1 complex formation, inhibits FGF2-induced Erk activation, and thereby suppresses FGF2-driven angiogenesis and tumor growth. Our work unveils a previously unrecognized new function of VEGF-B as an endogenous inhibitor of FGF2/FGFR1-driven functions.

### VEGF-B has an inhibitory effect on FGF2/FGFR1 signaling

Independent studies have reported anti-angiogenic and anti-tumor effects of VEGF-B.^8–11^ For example, in various mouse models, VEGF-B inhibited tumor angiogenesis and tumor growth.^9,10^ In humans, high VEGF-B levels are associated with low tumor angiogenesis, better survival, and low risks of cancer,^8,11,12^ and low VEGF-B levels high tumor angiogenesis, poor survival, and high risks of cancer,^8,11,13^ further advocating anti-angiogenic and anti-tumor effects of VEGF-B. Notwithstanding, since the mechanisms underlying these observations are unclear, these critical findings have long been ignored.

In this work, we discovered that VEGF-B acts as an inhibitor of FGF2/FGFR1 when they are abundantly expressed. Indeed, FGF2 and FGFR1 are frequently up-regulated in tumors and play important roles in tumor angiogenesis and tumor growth.^38^ Therefore, in tumors with high FGF2/FGFR1 levels, VEGF-B deficiency could result in greater tumor angiogenesis and tumor growth driven by FGF2/FGFR1. This may be particularly relevant for breast cancer, since FGFR1 amplification is found in 16–27% of luminal B-type breast cancers.^39^ Given the inhibitory effect of VEGF-B on FGF2/FGFR1, high VEGF-B levels may be beneficial for breast cancer patients by damping FGF2/FGFR1-induced tumor neovasculature and tumor growth.^11^ Indeed, blocking VEGF-B (together with other VEGF family members) has failed to show any benefit for most types of cancers.^14,15^ As such, at least one possible explanation among others might be the loss of VEGF-B’s inhibition on FGF2/FGFR1 signaling in the tumors treated with VEGF-B-blocking drugs.

### When is VEGF-B anti- or pro-angiogenic?

Given that VEGF-B can be pro-^2,5,40,41^ or anti-angiogenic^8–10^ (and current study) depending on the specific context, outstanding questions thus exist. Under which conditions is VEGF-B anti-angiogenic, and under which pro-angiogenic? And, what controls this switch? Our current study shows that at least one of the determining factors is whether FGF2/FGFR1 are expressed and at what levels. That is - when FGF2/FGFR1 are highly expressed, VEGF-B can be anti-angiogenic by inhibiting the FGF2/FGFR1 pathway (Fig. 7e). However, when FGF2/FGFR1 are not expressed/low, VEGF-B thus has no/little FGF2/FGFR1 to inhibit but can be pro-angiogenic by promoting blood vessel survival as reported previously by our and other groups^2,4–7^ (Fig. 7f).

Indeed, multiple assays, such as EC migration/sprouting and tumor growth assays showed that when FGFR1/FGF2 were highly expressed or when FGF2 was administered, VEGF-B displayed an inhibitory effect, suggesting that under conditions of high FGFR1/FGF2 levels, VEGF-B can be anti-angiogenic by suppressing FGF2 function (Fig. 7e). FGFR1/FGF2 are highly expressed in developing mouse and rat retinae (Supplementary Fig. 17a, e)^42,43^ and many types of tumors,^38,39^ where the anti-angiogenic effect of VEGF-B has been observed (Supplementary Table 1). On the other hand, the previously reported pro-angiogenic effect of VEGF-B has been mostly found under conditions characterized by tissue/blood vessel degeneration, where no or low level of FGFR1 is present due to cell/tissue death, such as in myocardial infarction or degenerated retina (Supplementary Fig. 17b–f)^5,40,41^ or blood vessel regression after FGF2 withdrawal.^2^ Consistently, other groups have also reported that FGFR1 levels are much lower in adult mouse/rat hearts (almost nine folds lower than in embryonic hearts).^44,45^

We have previously shown that VEGF-B is pro-angiogenic in mouse models of choroidal neovascularization and retinopathy of prematurity.^2^ Notably, these two models are, in fact, more alike vascular regression models since the neovasculature in these models eventually regresses nearly completely.^46–48^ Therefore, under such conditions of vascular degeneration, the previously reported anti-apoptotic effects of VEGF-B^6,7^ can take place and increase blood vessel survival, as such, acting pro-angiogenic (Fig. 7f). In summary, depending on the specific condition (e.g., high *versus* low/no FGF2/FGFR expression), the vascular function of VEGF-B can be anti- or pro-angiogenic (Fig. 7e, f).

### Mechanism underlying the inhibitory effect of VEGF-B on FGF2/FGFR1 pathway

Our data show that the inhibitory effect of VEGF-B may be mediated by its induction of VEGFR1/FGFR1 complex formation. Baseline levels of VEGFR1/FGFR1 complex were detected in multiple mouse tissues, and VEGF-B treatment increased this complex formation in both cultured endothelial cells and mouse retinae. Noteworthy, VEGF-B binds to VEGFR1/FGFR1 with a higher affinity (K_D_ = 1.4 nM) than that for FGFR1/FGFR1 (K_D_ = 17 nM), suggesting that the inhibitory effect of VEGF-B may be mainly through VEGFR1/FGFR1 complex formation. Indeed, genetic studies showed that VEGF-B inhibited FGF2-induced Erk activation in wild-type ECs with *Flt1* and *Fgfr1* expression but not in ECs with loss of either *Flt1* or *Fgfr1*, thus supporting a critical requirement of VEGFR1/FGFR1 complex in mediating the inhibitory effect of VEGF-B. Consistently, VEGFR1 has been shown to have an anti-angiogenic effect under many conditions by inhibiting Erk activation,^20,21^ which could contribute to the inhibitory effect of VEGF-B-induced VEGFR1/FGFR1 complex compared with FGF2-induced FGFR1/FGFR1 homodimer.

### Possible translational implications

Our findings may have translational relevance. Currently, drugs that block VEGF-B together with other VEGF family members are being used in the clinic to treat patients with various neovascular diseases.^14^ Given the anti-angiogenic nature of VEGF-B under conditions of high FGF2/FGFR1 levels, caution is warranted to inhibit VEGF-B indiscriminately when treating patients with neovascular diseases.

## Materials and methods

Other parts of Materials and Methods are included in the Supplementary Information.

### Surface plasmon resonance assay for FGFR1 or VEGFR1 binding

Human VEGF-B_167_ (100-20B, PeproTech) was used throughout this study and is referred to as VEGF-B unless specified otherwise. VEGF-B binding to FGFR1 was tested using a surface plasmon resonance assay using a BIAcore 8000 system (GE Healthcare). Specifically, VEGF-B (PeproTech), FGF2 (BFF-H4117, Acro Biosystems), PlGF (100-06, PeproTech) were immobilized at 5389, 1860, or 4700 resonance units (RU), respectively, onto a CM5 (carboxymethylated dextran matrix) sensor chip using an amine coupling kit (Biacore). The unreactive groups on the chip were blocked by ethanolamine according to the manufacturer’s instructions. A continuous flow (30 µl/min) of serially diluted FGFR1-Fc (12.5, 25, 50, 100, 200, and 400 nM, Z03223, Genscript) or VEGFR1-Fc (3.125, 6.25, 12.5, 25, 50, and 100 nM, R&D Systems) onto the immobilized ligand surface was monitored by passing the analytes across the sensor chip. The sensor surface was regenerated between assays by 60 sec of treatment with 10 mM of glycine (pH 1.5 and 2.0). The BIAevaluation software (Biacore) was used for analyses. Kinetic constants were obtained from the sensorgrams using a BIAcore 8000 evaluation software with a 1:1 binding model (Biacore). Dissociation constant (K_D_*)* was calculated from the ratio of the dissociation and association rate constants (K_D_ *= kd/ka)*.

To test which extracellular domains of FGFR1 VEGF-B binds to, VEGF-B protein (PeproTech) was immobilized at 3820 RU onto a CM5 chip. A continuous flow (10 µl/min) of serially diluted proteins (FGFR1 DI: 18.75, 37.5, 75, 150, 300, 600 nM; FGFR1 DII: 3.125, 6.25, 12.5, 25, 50, 100 nM; FGFR1 DIII: 12.5, 25, 50, 100, 200, 400 nM; FGFR1 DII-III: 25, 50, 100, 200, 400, 800 nM) onto the immobilized VEGF-B surface was monitored by passing the analytes across the sensor chip.

To test whether VEGF-B competes with FGF2 for FGFR1 binding, VEGF-B protein (PeproTech) was immobilized at 6900 RU onto a CM5 chip using an amine coupling kit. A continuous flow (10 µl/min) of FGFR1-Fc (200 nM, Genscript) with 6.25 nM of BSA, FGF2 (PeproTech) or PlGF (PeproTech), respectively, onto the surface with immobilized VEGF-B was monitored by passing the analytes across the sensor chip.

To test whether VEGF-B binds to the VEGFR1/FGFR1 or FGFR1/VEGFR1 heterodimers, VEGF-B protein (PeproTech) was immobilized at 3820 RU onto a CM5 chip. A continuous flow (10 µl/min) of serially diluted heterodimer proteins (1.875, 3.75, 7.5, 15, 30, 60 nM) onto the immobilized VEGF-B surface was monitored. The sensor surface was regenerated between assays by 60 s of treatment with 10 mM of glycine (pH 2.0). The BIAevaluation software (Biacore) was used for the interaction analyses.

### VEGF-B synthetic peptides and FGFR1 binding analysis

Biotinylated human VEGF-B_167_ (UnitPro ID: P49765) peptides (Table 1) were synthesized and purified using high pressure liquid chromatography (HPLC, Genscript). The sequences of the peptides were verified by matrix-assisted laser desorption ionization-time of flight mass spectrometry.

To test whether the VEGF-B peptides binds to FGFR1, an ELISA assay was used and Fc specific anti-human IgG antibody (I2136, Sigma) in PBS was captured onto a 96-well plate (Nunc maxisorp flat-bottom, 44-2404-21, Invitrogen) overnight at 4 °C followed by blocking with 1% BSA in PBS for 1 h. After incubation for 1 h with 20 nM of FGFR1-Fc protein (Z03223, Genscript), biotinylated FGF2 (200 nM, BFF-H4117, Acro Biosystems) and biotinylated VEGF-B peptides (200 nM, Genscript) were added to the wells and incubated for 2 h. The plates were washed with PBS followed by incubation with an HRP-conjugated Streptavidin antibody (N100, Thermo Scientific) for 1 h. Binding signals were developed using the 1-Step TMB ELISA solution (Thermo Scientific) and the optical densities measured at 450 nm.

### Competitive FGFR1 binding assay using ELISA

First, a standard curve of FGF2 binding to FGFR1 was generated. The plates were coated with FGF2 protein (2.5 µg/ml, PeproTech) for overnight. The wells were washed and blocked with 1% BSA in PBS. Then, serially diluted FGFR1-Fc protein (2 nM to 0.03125 nM, 658-FR, R&D Systems) in PBS containing 1% BSA was incubated in the FGF2-coated wells for 30 min. The wells were washed and incubated with anti-human IgG-HRP to detect the bound FGFR1-Fc using the 1-Step Ultra TMB (3,3',5,5'-tetramethylbenzidine)-ELISA substrate solution (34028, Thermo Scientific). The absorbance was measured at 450 nm. The data points were fitted by saturation binding using a GraphPad Prism software (San Diego, CA). To test whether VEGF-B competes with FGF2 for FGFR1 binding, serially diluted (3.125, 6.25, 12.5, 25, 50, and 100 nM) VEGF-B (PeproTech) or PlGF (100-06, PeproTech) proteins were incubated with FGFR1-Fc (0.4 nM) for 1 h prior to incubation in the FGF2-coated wells. The wells were then washed and incubated with anti-human IgG-HRP to detect bound FGFR1-Fc using the 1-Step Ultra TMB-ELISA substrate solution (Thermo Scientific). The absorbance was measured at 450 nm. The amount of FGFR1-Fc bound to FGF2 after competition with VEGF-B or PlGF was normalized using the generated standard curve of FGF2 binding to FGFR1.

### Cell culture

Primary human umbilical vein endothelial cells (HUVECs, 8000, ScienCell, Carlsbad, CA), human retinal endothelial cells (HRECs, HZ-H1095, HZbscience, Shanghai, China), and human dermal microvascular endothelial cell 1 (HMEC1, ZQ0456, ZQXZBIO, Shanghai, China, authenticated by STR profiling) were cultured in endothelial cell medium (ECM, ScienCell Research, Carlsbad, CA) with endothelial cell growth supplement (ECGS) and 5% FCS (ScienCell). The endothelial cells within eight passages were verified by Von Willebrand factor or CD31 staining and used for experiments. Hela cells (ZQ0068, ZQXZBIO, Shanghai, China, authenticated by STR profiling) were grown in DMEM, supplemented with 10% FBS, 100 U/ml penicillin, and 100 µg/ml streptomycin. Mycoplasma contamination was not found by PCR analysis.

### In situ proximity ligation assay (PLA)

The PLA assays were performed using a DuolinkII PLA kit (DUO92007, Sigma) according to the manufacturer’s instruction. HRECs were stimulated with VEGF-B or PlGF (50 ng/ml each, PeproTech) for 30 min and then fixed with 4% paraformaldehyde. The cells were permeabilized with 0.5% TX-100 in PBS for 15 min. To visualize protein–protein complexes, rabbit anti-FGFR1 (9740, Cell Signaling Technology) and mouse anti-VEGFR1 (10136-MM03, Sino Biological) antibodies were used and followed by Duolink II anti-mouse plus and Duolink II anti-rabbit minus secondary antibodies (DUO92005, Sigma). The images were analyzed using an ImageJ program (NIH, Bethesda, MD).

### Phospho-MAPK antibody array screening and Erk activation

For phospho-MAPK antibody array screening, sub-confluent human dermal microvascular endothelial cell 1 (HMEC1, BNCC338511, BeNa Culture Collection, Beijing, China) were starved in serum-free medium for 6 h and treated with 50 ng/ml human VEGF-B (PeproTech) for 30 min, washed with ice-cold PBS, and lysed in lysis buffer provided by the human phospho-kinase array kit (ARY003B, R&D Systems). Protein concentrations were determined using a micro BCA protein assay kit (Pierce). The antibody array membrane (ARY003B, R&D Systems) was incubated in the array buffer 1 for 1 h at room temperature, followed by incubation of the array membrane with the cell extracts at 4 °C for overnight, and then washed with wash buffer and incubated with the detection antibody cocktail for 2 h at room temperature. The array membrane was washed with wash buffer, followed by incubation with Streptavidin-HRP for 30 min at room temperature.

For Erk activation assay, sub-confluent ECs were starved in serum-free medium for 6 h and then treated with human FGF2 (50 ng/ml, PeproTech) or VEGF-B (100 ng/ml, PeproTech) for 10 or 30 min as indicated in the figure legends. Cell lysates were prepared using RIPA lysis buffer with protease and phosphatase inhibitors and subjected to Western blot. Immunoreactivity was visualized using the enhanced chemiluminescence reagent (ECL, Pierce), scanned using a G:Box device (Syngene, Frederick, MD, USA) and images analyzed using an ImageJ program (NIH, Bethesda, MD, USA).

### Animal care and use

All animal experiments were approved by the animal research ethics committees at the Sun Yat-Sen University. All animals were handled in accordance with the approved guidelines. Detailed information on *Fgfr1*^*lox/lox*^, *Flt1*^*lox/lox*^, *rd1*/*rd1*, wild-type C57BL/6 mice and *Vegf-b*^*−/−*^ mice are described in Supplementary Information.

### In vivo Erk activation in mouse retinae

For in vivo Erk activation, BSA (500 ng/eye, Sigma), VEGF-B (500 ng/eye, PeproTech), FGF2 (100 ng/eye, PeproTech), VEGF-B (500 ng/eye) + FGF2 (100 ng/eye), VEGF-A (100 ng/eye, 100-20, PeproTech), or VEGF-B (500 ng/eye) + VEGF-A (100 ng/eye) were intravitreally injected into 8-week-old mouse eyes. After 30 min, the retinae were harvested, and Western blots were performed to check Erk phosphorylation.

### In vivo Matrigel assay

The Matrigel assay was performed as described previously.^49^ Briefly, two aliquots (0.5 ml) of growth factor-reduced Matrigel (BD Bioscience, 356230) supplemented with BSA (150 ng/ml, Sigma), human FGF2 (150 ng/ml, PeproTech), human VEGF-B (300 ng/ml, PeproTech), human PlGF (300 ng/ml, PeproTech), FGF2 (150 ng/ml) + VEGF-B (300 ng/ml), or FGF2 (150 ng/ml) + PlGF (300 ng/ml), respectively, were injected subcutaneously into the mid-abdominal region of C57Bl6 adult mice. After 7 days, the mice were euthanized. The Matrigel plugs were harvested, fixed with 4% PFA, and processed for sections and CD31 (Abcam, ab222783) staining. Each Matrigel plug was sectioned throughout and all the sections collected. For each plug, five sections distributed evenly throughout the plug (from the beginning to the end) were analyzed. Images were collected using a Zeiss axiovert 200M microscope equipped with a 20x plan-apochromat (N.A. 0.5) objective lens and axiocam MRc5 CCD camera using an Axiovision image acquisition software (v.4.6) (Zeiss MicroImaging). For angiogenesis analysis, threshold parameters for angiogenesis in the Matrigels were defined by cell density (red color of CD31 staining) using a Meta Morph software (ver 6.1, Molecular Devices). The threshold areas corresponding to the vascular areas were measured using a “region measurement” function. The vascular areas were presented as the ratio of the vascular pixels versus total pixels per microscopic field.

### Immunofluorescence staining

For immunostaining, cryosections of retinae were fixed in 4% PFA for 20 min, permeabilized with 0.3% Triton X-100 in PBS for 5 min, and blocked with 1% BSA in PBS for 1 h at room temperature. The sections were then incubated with primary antibodies overnight at 4 °C. The corresponding secondary antibodies were then incubated for 1 h. DAPI (D3571, Life Technologies) was used for nuclear staining. The primary antibody used was anti-CD31 (553370, BD Bioscience). The secondary antibodies and other reagents used were Alexa fluor 555-conjugated anti-rabbit antibody (A31572, Life Technologies), Alexa 555-conjugated anti-rat antibody (A21434, Life Technologies). Images were obtained and analyzed using a Zeiss microscope (LSM710).

### Statistics and data analysis

GraphPad Prism 8.2.1 or IBM SPSS statistic V25 were used to generate graphs and perform statistical analysis. *P* < 0.05 were considered statistically significant. More details are described in Supplementary Information.

### Supplementary information

instructions for supplementary information

Supplementary information

## Data Availability

All the data required to evaluate this work are present in the paper and the related supplementary information. Requests for resources and further information should be directed to Xuri Li (lixr6@mail.sysu.edu.cn).

## References

[1] Nag S, Eskandarian MR, Davis J, Eubanks JH (2002). Differential expression of vascular endothelial growth factor-A (VEGF-A) and VEGF-B after brain injury. J. Neuropathol. Exp. Neurol..

[2] Zhang F (2009). VEGF-B is dispensable for blood vessel growth but critical for their survival, and VEGF-B targeting inhibits pathological angiogenesis. Proc. Natl Acad. Sci. USA.

[3] Robciuc MR (2016). VEGFB/VEGFR1-induced expansion of adipose vasculature counteracts obesity and related metabolic complications. Cell Metab..

[4] Kivela R (2019). Endothelial cells regulate physiological cardiomyocyte growth via VEGFR2-mediated paracrine signaling. Circulation.

[5] Li X (2008). Reevaluation of the role of VEGF-B suggests a restricted role in the revascularization of the ischemic myocardium. Arterioscler Thromb. Vasc. Biol.

[6] Rasanen M (2016). VEGF-B gene therapy inhibits doxorubicin-induced cardiotoxicity by endothelial protection. Proc. Natl Acad. Sci. USA.

[7] Li Y (2008). VEGF-B inhibits apoptosis via VEGFR-1-mediated suppression of the expression of BH3-only protein genes in mice and rats. J. Clin. Invest.

[8] Brouwer NJ (2019). Tumour angiogenesis in uveal melanoma is related to genetic evolution. Cancers.

[9] Albrecht I (2010). Suppressive effects of vascular endothelial growth factor-B on tumor growth in a mouse model of pancreatic neuroendocrine tumorigenesis. PLoS ONE.

[10] Yang X (2015). VEGF-B promotes cancer metastasis through a VEGF-A-independent mechanism and serves as a marker of poor prognosis for cancer patients. Proc. Natl Acad. Sci. USA.

[11] Zajkowska M, Lubowicka E, Malinowski P, Szmitkowski M, Lawicki S (2018). Plasma levels of VEGF-A, VEGF B, and VEGFR-1 and applicability of these parameters as tumor markers in diagnosis of breast cancer. Acta Biochim. Pol..

[12] Baty F (2010). Gene profiling of clinical routine biopsies and prediction of survival in non-small cell lung cancer. Am. J. Respir. Crit. Care Med..

[13] Sanmartin E (2014). A gene signature combining the tissue expression of three angiogenic factors is a prognostic marker in early-stage non-small cell lung cancer. Ann. Surg. Oncol..

[14] Ricci V, Ronzoni M, Fabozzi T (2015). Aflibercept a new target therapy in cancer treatment: a review. Crit. Rev. Oncol. Hematol..

[15] Apte RS, Chen DS, Ferrara N (2019). VEGF in signaling and disease: beyond discovery and development. Cell.

[16] Olofsson B (1998). Vascular endothelial growth factor B (VEGF-B) binds to VEGF receptor-1 and regulates plasminogen activator activity in endothelial cells. Proc. Natl Acad. Sci. USA.

[17] Ho VC, Duan LJ, Cronin C, Liang BT, Fong GH (2012). Elevated vascular endothelial growth factor receptor-2 abundance contributes to increased angiogenesis in vascular endothelial growth factor receptor-1-deficient mice. Circulation.

[18] Lebok P (2016). Loss of membranous VEGFR1 expression is associated with an adverse phenotype and shortened survival in breast cancer. Mol. Med. Rep..

[19] Lohri C (2014). Neutrophil expression of ICAM1, CXCR1, and VEGFR1 in patients with breast cancer before and after adjuvant chemotherapy. Anticancer Res..

[20] Szabo E (2016). Autocrine VEGFR1 and VEGFR2 signaling promotes survival in human glioblastoma models in vitro and in vivo. Neuro. Oncol.

[21] Zhang Z, Neiva KG, Lingen MW, Ellis LM, Nor JE (2010). VEGF-dependent tumor angiogenesis requires inverse and reciprocal regulation of VEGFR1 and VEGFR2. Cell Death Differ..

[22] Nicoli S, De Sena G, Presta M (2009). Fibroblast growth factor 2-induced angiogenesis in zebrafish: the zebrafish yolk membrane (ZFYM) angiogenesis assay. J. Cell Mol. Med..

[23] Welti JC (2011). Fibroblast growth factor 2 regulates endothelial cell sensitivity to sunitinib. Oncogene.

[24] Cao R (2004). Comparative evaluation of FGF-2-, VEGF-A-, and VEGF-C-induced angiogenesis, lymphangiogenesis, vascular fenestrations, and permeability. Circ. Res..

[25] Hoppenreijs VP, Pels E, Vrensen GF, Treffers WF (1994). Basic fibroblast growth factor stimulates corneal endothelial cell growth and endothelial wound healing of human corneas. Invest. Ophthalmol. Vis. Sci..

[26] Biro S (1994). Expression and subcellular distribution of basic fibroblast growth factor are regulated during migration of endothelial cells. Circ Res..

[27] Amann K (2006). Impaired myocardial capillarogenesis and increased adaptive capillary growth in FGF2-deficient mice. Lab. Invest..

[28] Rousseau B, Larrieu-Lahargue F, Bikfalvi A, Javerzat S (2003). Involvement of fibroblast growth factors in choroidal angiogenesis and retinal vascularization. Exp. Eye Res..

[29] Oladipupo SS (2014). Endothelial cell FGF signaling is required for injury response but not for vascular homeostasis. Proc. Natl Acad. Sci. USA.

[30] Bono F (2013). Inhibition of tumor angiogenesis and growth by a small-molecule multi-FGF receptor blocker with allosteric properties. Cancer Cell.

[31] Marzioni D (2009). Expression of basic fibroblast growth factor, its receptors and syndecans in bladder cancer. Int. J. Immunopathol. Pharmacol..

[32] Armstrong K (2011). Upregulated FGFR1 expression is associated with the transition of hormone-naive to castrate-resistant prostate cancer. Br. J. Cancer.

[33] Sasaki H (2012). Increased FGFR1 copy number in lung squamous cell carcinomas. Mol. Med. Rep..

[34] Li X, Aase K, Li H, von Euler G, Eriksson U (2001). Isoform-specific expression of VEGF-B in normal tissues and tumors. Growth Factors.

[35] Ornitz DM, Itoh N (2015). The fibroblast growth factor signaling pathway. Wiley Interdiscip. Rev. Dev. Biol..

[36] Leonard P (2008). Crystal structure of vascular endothelial growth factor-B in complex with a neutralising antibody Fab fragment. J. Mol. Biol..

[37] Haugsten EM, Sorensen V, Brech A, Olsnes S, Wesche J (2005). Different intracellular trafficking of FGF1 endocytosed by the four homologous FGF receptors. J. Cell Sci..

[38] Presta M, Chiodelli P, Giacomini A, Rusnati M, Ronca R (2017). Fibroblast growth factors (FGFs) in cancer: FGF traps as a new therapeutic approach. Pharmacol. Ther..

[39] Turner N (2010). FGFR1 amplification drives endocrine therapy resistance and is a therapeutic target in breast cancer. Cancer Res..

[40] Lahteenvuo JE (2009). Vascular endothelial growth factor-B induces myocardium-specific angiogenesis and arteriogenesis via vascular endothelial growth factor receptor-1- and neuropilin receptor-1-dependent mechanisms. Circulation.

[41] Tirziu D (2007). Myocardial hypertrophy in the absence of external stimuli is induced by angiogenesis in mice. J. Clin. Invest..

[42] Clark BS (2019). Single-cell RNA-seq analysis of retinal development identifies NFI factors as regulating mitotic exit and late-born cell specification. Neuron.

[43] Prokosch-Willing V, Meyer zu Hoerste M, Mertsch S, Stupp T, Thanos S (2015). Postnatal visual deprivation in rats regulates several retinal genes and proteins, including differentiation-associated fibroblast growth factor-2. Dev. Neurosci..

[44] Jin Y (1994). Cloning and expression of fibroblast growth factor receptor-1 isoforms in the mouse heart: evidence for isoform switching during heart development. J. Mol. Cell Cardiol..

[45] Liu L (1995). Adult cardiomyocytes express functional high-affinity receptors for basic fibroblast growth factor. Am. J. Physiol..

[46] Giani A (2011). In vivo evaluation of laser-induced choroidal neovascularization using spectral-domain optical coherence tomography. Invest. Ophthalmol. Vis. Sci..

[47] Hisatomi T (2012). The regulatory roles of apoptosis-inducing factor in the formation and regression processes of ocular neovascularization. Am. J. Pathol..

[48] Connor KM (2009). Quantification of oxygen-induced retinopathy in the mouse: a model of vessel loss, vessel regrowth and pathological angiogenesis. Nat. Protoc..

[49] Economopoulou M (2009). Histone H2AX is integral to hypoxia-driven neovascularization. Nat. Med..

