# Supplementary material for: VEGF-B prevents excessive angiogenesis by inhibiting FGF2/FGFR1 pathway
Source: Signal Transduct Target Ther. 2023 Aug 18;8:305. doi: 10.1038/s41392-023-01539-9 (PMC10435562; doi:10.1038/s41392-023-01539-9)
Supplement: Supplementary file 2 — Supplementary information [file 41392_2023_1539_MOESM2_ESM.docx]

Supplementary Materials for

**VEGF-B prevents excessive angiogenesis by inhibiting FGF2/FGFR1 pathway**

Chunsik Lee^1#^, Rongyuan Chen^1#^, Guangli Sun^2^, Xialin Liu^1^, Xianchai Lin^1^, Chang He^1^, Liying Xing^1^, Lixian Liu^1^, Lasse Jensen^3^, Anil Kumar^1^, Harald F. Langer^4^, Xiangrong Ren^1^, Jianing Zhang^1^, Lijuan Huang^1^, Xiangke Yin^1^, Jong Kyong Kim^1^, Juanhua Zhu^1^, Guanqun Huang^1^, Jiani Li^1^, Weiwei Lu^1^, Wei Chen^1^, Juanxi Liu^1^, Jiaxin Hu^1^, Qihang Sun^1^, Weisi Lu^1^, Lekun Fang^5^, Shasha Wang^1^, Haiqing Kuang^1^, Yihan Zhang^6^, Geng Tian^7^, Jia Mi^7^, Bi-Ang Kang^8^, Masashi Narazaki^9^, Aaron Prodeus^10^, Luc Schoonjans^11,12^, David M. Ornitz^13^, Jean Gariepy^10^, Guy Eelen^11,12^, Mieke Dewerchin^11,12^, Yunlong Yang^14^, Jing-Song Ou^8^, Antonio Mora^15^, Jin Yao^2^,

Chen Zhao^6*^, Yizhi Liu^1^, Peter Carmeliet^11,12^, Yihai Cao^16*^ & Xuri Li^1*^

Correspondence to: Yihai Cao, [Yihai.Cao@ki.se](mailto:Yihai.Cao@ki.se)

Xuri Li, [lixr6@mail.sysu.edu.cn](mailto:lixr6@mail.sysu.edu.cn)

Chen Zhao, [dr_zhaochen@163.com](mailto:dr_zhaochen@163.com)

^#^ Contributed equally

**This PDF file includes:**

Materials and Methods

Figures. S1 to S17

Tables S1 to S5

Materials and Methods

**FGFR1 and VEGFR1 recombinant proteins**

For the recombinant proteins of FGFR1 extracellular domain (ECD) I (FGFR1 DI), FGFR1 DII, FGFR1 DIII, FGFR1 DI-II, FGFR1 DII-III, the coding sequences were synthesized with codon optimization without changing the amino acid sequences (Detai Biotechnology, Nanjing, China). All the recombinant FGFR1 ECDs begin with a signal peptide and end with an Fc tag

(forward primer: 5’-CAAGTTTAAACGGATCTCTAGCGAATTCCCGCCGCCACCATGTGGGGTTGGAAGTGCCTGCTGTTTTGGGCC-3’,  reverse primer: 5’-GCCGGCCTCGAGCGGCCGCTAGCAAGCTTATCACTTGCCTGGGCTCAGGCTCAGGCTCTTCTGGGTGTAGT-3’).

For the recombinant proteins of VEGFR1 extracellular domain (ECD)-L-FGFR1 ECD-His (Supplementary Table 4), FGFR1 ECD-L-VEGFR1 ECD-His (Supplementary Table 5), FGFR1 cDNA plasmid was provided by Dr. Makoto Kuro-O at University of Texas Southwestern Medical Center, Dallas, TX, USA. VEGFR1 cDNA (HG10136-CF) was purchased from Sino Biological (Beijing, China). The respective DNA templates were generated using a MaxCodon Optimization Program (version 13, Detai Biotechnology, Nanjing, China). The DNA templates were subsequently cloned into the expression vector proEM (Detai Biotechnology), and each expression vector was verified by sequencing. The primers used are listed in Supplementary Table 2. The expression plasmids were transfected into HEK-293-6e cells (Detai Biotechnology) and the fusion proteins purified by Fc affinity chromatography. Coomassie blue staining was used to verify the purity of the recombinant proteins. Only proteins with a purity higher than 90% were used for experiments and were also verified by Western blot using an anti-Fc antibody (A01854-200, Genscript) or an anti-His antibody (MY1901, Merrybio).

**Antibodies used in Western blots**

For Western blots, the antibodies used were: anti β-actin antibody (RM2001, Ray Antibody Biotech), anti-tubulin antibody (RM2007, Ray Antibody Biotech), anti-phospho-Tyr 653 FGFR1 antibody (ab173305, Abcam), anti-phospho-Tyr 653/654 FGFR1 antibody (PA5-85806, Thermo Fisher Scientific), anti-VEGFR1 antibody (2893, Cell Signaling Technology), anti-FGF2 antibody (05-118, Millipore), anti-FGFR1 antibody (9740, Cell Signaling Technology), anti-VEGFR1 antibody (MA5-35280, Thermo Scientific) anti-Fc antibody (A01854-200, Genscript) and anti-His antibody (MY1901, Merrybio).

**Immunoprecipitation of VEGFR1/FGFR1 complex using mouse tissues**

To detect VEGFR1/FGFR1 complex using co-immunoprecipitation, mouse tissues were homogenized in RIPA lysis buffer (R0010, Solarbio, Beijing, China) with a protease and phosphatase inhibitor tablet (88668, Thermo Scientific). The tissue lysates were incubated with an anti-FGFR1 antibody (9740, Cell Signaling Technology) or control IgG (2729S, Cell Signaling Technology) for overnight at 4°C and precipitated using protein A/G plus agarose beads (sc-2003, Santa Cruz). Immunoprecipitated samples were separated using an 8% SDS-PAGE gel and transferred to a PVDF membrane. The membranes were incubated with the following antibodies respectively: anti-VEGFR1 antibody (MA5-35280, ThermoFisher Scientific) and anti-FGFR1 antibody (9740, Cell Signaling Technology). Immunoreactivity was detected using a horseradish-peroxidase (HRP)-conjugated secondary antibody (Multisciences Biotech, Hangzhou, China) and visualized using enhanced chemiluminescence (WBKLS0500, Millipore). For *in vivo* VEGFR1 and FGFR1 complex detection in mouse retinae, human VEGF-B (500 ng/eye, PeproTech), human PlGF (500 ng/eye, PeproTech) or BSA (500 ng/eye, Sigma) were intravitreally injected into 8-week old mouse eyes. After two hours, the retinae were harvested for co-immunoprecipitation and immunoblotting analysis as described above.

**Immunoprecipitation of VEGFR1/FGFR1 complex using FGFR1 mutants in HUVECs**

To examine the effects of FGFR1 tyrosine residues Y463, Y585 and Y653 on VEGF-B-induced VEGFR1/FGFR1 complex formation, immunoprecipitation assays were performed using wild-type FGFR1 (FGFR1 WT) or different FGFR1 mutants using HUVECs. Briefly, adenovirus carrying the V5-taged wild-type FGFR1 (FGFR1 WT, NM_023110) or different FGFR1 mutants with tyrosine residues 463, 585 or 653 replaced with phenylalanine respectively (FGFR1 Y463F, FGFR1 Y585F and FGFR1 Y653F, Vigene Biosciences, Shandong, China) were used for experiments. HUVECs were infected with FGFR1 WT, FGFR1 Y463F, FGFR1 Y585F or FGFR1 Y653F respectively for 48 hours with a multiplicity of infection of 10. The HUVECs were then starved in serum-free medium for 4 hours and incubated with VEGF-B (100 ng/ml, PeproTech), human PlGF (100 ng/ml, PeproTech) or BSA (100 ng/ml, Sigma) respectively for 10 minutes followed by immunoprecipitation. The cell lysates were incubated with anti-V5 magnetic beads (P2141, Beyotime) for 2 hours at room temperature and the precipitated samples analyzed by Western blots.

***In vivo* *in situ* proximity ligation assay (PLA) in mouse retinae**

To detect VEGFR1/FGFR1 complex formation *in vivo*, human VEGF-B (500 ng/eye, PeproTech), human PlGF (500 ng/eye, PeproTech) or BSA (500 ng/eye, Sigma) were intravitreally injected into the eyes of 8-week old mice respectively. After two hours, the eyes were harvested, fixed using 4% paraformaldehyde, embedded in OCT and sectioned for PLA staining using a DuolinkII PLA kit (DUO92007, Sigma) according to the manufacturer’s instructions. The sections were permeabilized using 0.5% Triton-100 in PBS for 15 min. To visualize the protein-protein complexes, rabbit anti-FGFR1 (9740, Cell Signaling Technology) and/or mouse anti-VEGFR1 (10136-MM03, Sino Biological) antibodies were used followed by the Duolink II anti-mouse plus and Duolink II anti-rabbit minus secondary antibodies (DUO92005, Sigma). The images were analyzed using the ImageJ program (NIH, Bethesda, MD).

**Site-directed mutagenesis of FGFR1**

The cDNA encoding mouse FGFR1c in pcDNA 3.1 (+) vector was kindly provided by Dr. Makoto Kuro-o (University of Texas Southwestern Medical Center, Dallas, TX) and was sub-cloned into a pEZ-LV201 vector (GeneCopoeia). Site-directed mutagenesis was performed using a Quickchange lighting site-directed mutagenesis kit (Agilent Technologies) according to the manufacturer’s instructions. The point mutations of tyrosine (Y) to phenylalanine (F) at different tyrosine sites (FGFR1 Y463, Y583, Y585, Y653, Y654, and Y766) were introduced with oligonucleotides (supplementary Table 3). The expression vectors were verified by sequencing. The mutated FGFR1 receptors were expressed in HeLa cells, which have little endogenous FGFR1 expression ^1^, by transfection using Lipofectamine 2000 (Life Technologies) according to the manufacturer’s instructions. Two days after transfection, Western blots were performed to analyze Erk phosphorylation.

**Erk activation with/without heparin or neuropilin 1 (NRP1) knockdown**

For Erk activation assay with/without heparin, sub-confluent HRECs were starved in serum-free medium for four hours and incubated with 100 ng/ml of heparin (HY-17567, Medchem Express, Shanghai, China) for 30 min. For NRP1 knockdown, the HRECs were transfected with human NRP1 siRNA or non-targeting scrambled control siRNA (Riobio, Guangzhou, China) using the ESCORT transfection reagent (L3287, Sigma) for 48 hours before serum-free medium starvation. For grow factor stimulation, human FGF2 (30 ng/ml, PeproTech) or VEGF-B (100 ng/ml, PeproTech) were added to the cells and incubated for 10 minutes. Cell lysates were prepared using the RIPA lysis buffer with protease and phosphatase inhibitors and subjected to Western blot. Immunoreactivity was visualized using the enhanced chemiluminescence reagent (ECL, Pierce), scanned using a G:Box device (Syngene, Frederick, MD, USA), and images analyzed using the ImageJ program (NIH, Bethesda, MD, USA).

**Cell migration assay**

HUVECs or HRECs were seeded into 48-well plates to adhere overnight. When the cells were confluent, they were incubated with mitomycin C (5 µg/ml, HY13316, MedChem Express, Shanghai, China) for 30 minutes to arrest cell proliferation. The cells were wounded using a fine pipette-tip after cell-monolayer confluence and washed with PBS to remove the debris. Cells were treated with human FGF2 (50 ng/ml, PeproTech), human VEGF-B (100 ng/ml, PeproTech), human PlGF (100 ng/ml, PeproTech), FGF2 (50 ng/ml) + VEGF-B (100 ng/ml), or FGF2 (50 ng/ml) + PlGF (100 ng/ml) protein in serum-free medium for 24 hours. Migrated cells were quantified using an ImageJ program (NIH, Bethesda, MD).

To test the functionality of recombinant FGFR1 DII-III protein, HUVEC migration assay was performed. Confluent HUVECs were starved in serum-free medium for four hours. The cells were then wounded using a tip scraper, washed with serum-free medium, and incubated for seven hours in serum-free medium containing human FGF2 (30 ng/ml, PeproTech), FGF2 (30 ng/ml) + FGFR1 DII-III (100 ng/ml), or FGF2 (30 ng/ml) + FGFR1 DI-II (500 ng/ml) respectively. Each well was photographed at 10× magnification before and after treatment. Migrated cells were quantified using an ImageJ program (NIH, Bethesda, MD).

**Endothelial cell (EC) spheroid assay**

The EC spheroid assay was performed as described ^2^. Briefly, 3.2 x 10^5^ of HRECs or HUVECs (Angio-Proteomie, 800 cells/spheroid) were suspended in endothelial cell medium (ECM, ScienCell Research) containing 20% methylcellulose (M0512, Sigma) and seeded in round-bottom 96-well plates. EC spheroids were resuspended in collagen I (C3867, Sigma) with 50 ng/ml each of the following: human FGF2 (PeproTech), human PlGF (PeproTech), and human VEGF-B (PeproTech), FGF2 + PlGF, FGF2 + VEGF-B and BSA cultured for 24 hours. After 24 hours, the images were obtained and the EC sprouts and their total length were analyzed using an ImageJ program (NIH, Bethesda, MD).

***Flt1^lox/lox^* and *Fgfr1^lox/lox^* mice**

All animal experiments were approved by the Animal Care and Use Committee of Zhongshan Ophthalmic Center at the Sun Yat-sen University, Guangzhou, People’s Republic of China. All animals were handled in accordance with approved guidelines. The *Fgfr1 ^lox/lox^*, *Flt1 ^lox/lox^* and *rd1*/*rd1* mice were described previously ^3-5^ and were validated.

***Vegf-b^-/-^* mice**

*Vegf-b* knockout mice were generated by the Knockout Mouse Phenotyping Program (KOMP^2^) and were obtained from the Jackson Laboratory (*Vegfb^tm1b(EUCOMM)Hmgu^*, Bar Harbor, ME, USA). Briefly, the targeting vector is composed of an FRT site followed by a lacZ cassette and a loxP site (Supplementary Fig. 7a). The first loxP site is followed by a neomycin cassette, a second FRT site, and a second loxP site. A third loxP site is inserted downstream of the targeted exons (Supplementary Fig. 7a). The construct was introduced into C57Bl/6N-derived JM8.N4 embryonic stem (ES) cells. The correctly targeted ES cells were injected into B6(Cg)-Tyrc-2J/J (Stock No. 58) blastocysts. The resulting chimeric male mice were bred onto C57Bl/6NJ female mice and then to B6N.Cg-Tg (Sox2-Cre)1Amc/J mice (Stock No. 014094) to remove the floxed neomycin and targeted exon sequences, leading to the deletion of exons 2-6 (Supplementary Fig. 7a,b). The *Vegf-b* knockout mice were genotyped by PCR. The wild-type allele produces a fragment of 240 bp using the following primers: forward: 5’-GGG GAT CCT CAG TTC AAA CC-3’, reverse: 5’-CAG GCA TAC TCT GGG AAT GG-3’. The targeted allele generates a fragment of 450 bp using the following primers: forward: 5’- CGG TCG CTA CCA TTA CCA GT-3’, reverse: 5’- GCA GAT TGG AAA AAG CCT TC-3’. The *Vegf-b* knockout mice were bred on C57Bl6 background for more than six generations and littermates were used for experiments. Experiments were repeated at least twice.

**Isolation and culture of murine primary vascular endothelial cells**

Murine primary endothelial cells (ECs) were isolated from lungs of 6-8 weeks old mice. Lungs were harvested, minced and digested with collagenase type I (17018029, Life Technologies) at 37°C for 45 minutes. Sheep anti-rat IgG conjugated Dynabeads (11035, Life Technologies) were incubated with rat anti-mouse CD31 (553370, BD Biosciences) for overnight at 4°C and washed. The digested tissues were pelleted and resuspended in PBS with 0.1% BSA and incubated with rat anti-mouse CD31 conjugated Dynabeads at room temperature for 15 minutes. The bead-bound cells were recovered using a magnetic separator (CS15000, Invitrogen), washed, and resuspended in complete culture medium (DMEM containing 20% FBS, supplemented with 100 µg/ml heparin, 100 µg/ml endothelial cell growth factor growth supplement (Alfa Aesar, Ward Hill, MA), nonessential amino acids, L-glutamine and antibiotics. The cells were then plated into a gelatin-coated culture dish. Primary ECs within four passages were used for experiments.

**Gene deletion using adenovirus encoding the Cre enzyme (Cre-Ad) in primary ECs**

The Cre-expressing adenovirus (Cre-Ad, CV10010, Vigene Biosciences, Rockville, MD) was used to delete the floxed genes in primary mouse ECs as described previously ^6^. Briefly, the primary mouse ECs were infected with Cre-virus for 48 hours with a multiplicity of infection (MOI) of 10. Deletion of the genes of interest was verified by Western blot at 48 hours after virus infection.

**Phospho-receptor tyrosine kinase (pRTK) antibody array screening and FGFR1 activation**

For pRTK antibody array screening, sub-confluent human retinal endothelial cells (HRECs) were serum-free medium starved for six hours and treated with 50 ng/ml human VEGF-B (PeproTech) for 15 minutes. The cells were washed with ice-cold PBS and lysed in lysis buffer provided by the human RTK array kit (ARY001B, R&D Systems). Protein concentrations were determined using a micro BCA protein assay kit (Pierce). The antibody array membrane (ARY001B, R&D Systems) was incubated in the array buffer 1 for one hour at room temperature, followed by incubation of the array membrane with cell extracts at 4°C for overnight, and then washed with wash buffer and incubated with the anti-phospho-tyrosine-HRP antibody cocktail for two hours at room temperature. To investigate FGFR1 activation, sub-confluent HRECs were serum-free medium starved for six hours and treated with human VEGF-B (50 ng/ml, PeproTech) for 15 minutes at 37°C. Cell lysates were prepared using RIPA lysis buffer with a protease and phosphatase inhibitor tablet and subjected to Western blot. Immunoreactivity was visualized using the enhanced chemiluminescence (ECL, Pierce) and scanned using a G:Box device (Syngene, Frederick, MD, USA). The images were analyzed using an ImageJ program (NIH, Bethesda, MD, USA).

**Tumor formation assay**

The mouse fibrosarcarma cell line T241 cells with stable FGF2 expression (T241-FGF2) were described previously ^7^. For tumor formation assay, 2×10^6^ of T241-FGF2 cells in 100 µl PBS were subcutaneously injected into the middle region of the dorsal back of 6-8 weeks old *Vegf-b*^-/-^ mice or WT littermates. Tumor size was measured using a caliper (length×width^2^×π/6) ^8^. At day 15 after tumor cell implantation, the mice were sacrificed and the tumors harvested for all the analysis and staining. The tumor tissues were sectioned using a cryostat and sections fixed using 4% PFA for 15 minutes followed by CD31 (553370, BD Biosciences) immunofluorenscence staining according to the manufacturer’s instructions. The images of the whole tumor sections were taken using a Z2 Imager microscope (Carl Zeiss) and the CD31^+^ blood vessels analyzed using the ImageJ (NIH, Bethesda, MD) program in a blinded manner. Two-way ANOVA was used for statistics. Data are presented as mean ± s.e.m.

**Mouse myocardial infarction model**

For the mouse myocardial infarction model, 6-8 weeks old C57Bl6 mice were used and the left anterior descending coronary artery was ligated as described ^9^. 24 hours after ligation, the hearts were harvested and sectioned for immunofluorescence staining of FGFR1 (9740s, Cell Signaling Technology) and troponin (ZRB1355, Sigma, marker for heart infarction). Mice died within twenty-four hours of coronary artery ligation were considered as a technical failure and excluded from subsequent analysis.

**Statistics and data analysis**

Normal distribution of data was verified using Shapiro-Wilk, D'Agostino & Pearson, Kolmogorov-Smirnov test, or histogram using GraphPad Prism 8.2.1 or IBM SPSS statistic V25. In addition, when the sample size was small, the data normality was supported by evidence from literature reporting on analogous data sets ^10-13^. Data with normal distribution were analyzed using two-tailed *t*-test or ordinary ANOVA. Non-normally distributed data were analyzed using nonparametric tests. For comparisons of two groups, *t*-test was used for normally distributed data and Mann-Whitney test for not normally distributed data. For comparisons of ≥3 groups, two- or one-way ANOVA was used for data normally distributed and Kruskal-Wallis test for data not normally distributed. For multiple comparisons, *p* values were adjusted with post-hoc test including Sidak, Holm-Sidak, Dunn, Dunnett, two-stage linear step-up procedure of Benjamini, Krieger and Yekutieli, or LSD test. Difference was considered statistically significant when *p* or *q* (for multiple comparison adjustment, representing false discovery rate) < 0.05. The data are represented as mean ± s.e.m of the number of the determinations. All experiments using cultured cells were performed in triplicates. The sample sizes were chosen based on literature of others and our previous studies, in which the effects of VEGF-B, FGF2 and other related reagents have been demonstrated in similar model systems ^6,9,14-23^. Animal experiments were performed using littermates, otherwise they were assigned to match age and gender as much as possible. Experimental materials, including cell culture dishes and mouse littermates were randomly allocated into different experimental groups. Data analysis was performed in a blinded manner by a blinded individual whenever possible. Blinding was not employed in Western blots involving protein treatment (*i.e.*, FGF2 vs. VEGF-B, VEGF-A or PlGF) since the samples were loaded in specific orders for better data presentation. For Western blots, the intensities of the bands were determined using an ImageJ program (NIH, Bethesda, MD).

**References**

1 Haugsten, E. M., Sorensen, V., Brech, A., Olsnes, S. & Wesche, J. Different intracellular trafficking of FGF1 endocytosed by the four homologous FGF receptors. *J Cell Sci* **118**, 3869-3881 (2005).

2 Heiss, M. *et al.* Endothelial cell spheroids as a versatile tool to study angiogenesis in vitro. *FASEB J* **29**, 3076-3084 (2015).

3 Ho, V. C., Duan, L. J., Cronin, C., Liang, B. T. & Fong, G. H. Elevated vascular endothelial growth factor receptor-2 abundance contributes to increased angiogenesis in vascular endothelial growth factor receptor-1-deficient mice. *Circulation* **126**, 741-752 (2012).

4 Jacob, A. L., Smith, C., Partanen, J. & Ornitz, D. M. Fibroblast growth factor receptor 1 signaling in the osteo-chondrogenic cell lineage regulates sequential steps of osteoblast maturation. *Dev Biol* **296**, 315-328 (2006).

5 Otani, A. *et al.* Bone marrow–derived stem cells target retinal astrocytes and can promote or inhibit retinal angiogenesis. *Nat Med* **8**, 1004-1010 (2002).

6 Prost, S., Sheahan, S., Rannie, D. & Harrison, D. J. Adenovirus-mediated Cre deletion of floxed sequences in primary mouse cells is an efficient alternative for studies of gene deletion. *Nucleic Acids Res* **29**, E80 (2001).

7 Hosaka, K. *et al.* Dual roles of endothelial FGF-2-FGFR1-PDGF-BB and perivascular FGF-2-FGFR2-PDGFRbeta signaling pathways in tumor vascular remodeling. *Cell Discov* **4**, 3 (2018).

8 Rolny, C. *et al.* HRG inhibits tumor growth and metastasis by inducing macrophage polarization and vessel normalization through downregulation of PlGF. *Cancer Cell* **19**, 31-44 (2011).

9 Li, X. *et al.* Reevaluation of the role of VEGF-B suggests a restricted role in the revascularization of the ischemic myocardium. *Arterioscler Thromb Vasc Biol* **28**, 1614-1620 (2008).

10 Eilken, H. M. *et al.* Pericytes regulate VEGF-induced endothelial sprouting through VEGFR1. *Nat Commun* **8**, 1574 (2017).

11 Coppiello, G. *et al.* Meox2/Tcf15 heterodimers program the heart capillary endothelium for cardiac fatty acid uptake. *Circulation* **131**, 815-826 (2015).

12 Qian, Y. *et al.* TRIM47 is a novel endothelial activation factor that aggravates lipopolysaccharide-induced acute lung injury in mice via K63-linked ubiquitination of TRAF2. *Signal Transduct Target Ther* **7**, 148 (2022).

13 Li, X. *et al.* Lipid metabolism dysfunction induced by age-dependent DNA methylation accelerates aging. *Signal Transduct Target Ther* **7**, 162 (2022).

14 Aase, K. *et al.* Vascular Endothelial Growth Factor-B-Deficient Mice Display an Atrial Conduction Defect. *Circulation* **104**, 358-364 (2001).

15 Chang, L. K. *et al.* Dose-dependent response of FGF-2 for lymphangiogenesis. *Proc Natl Acad Sci U S A* **101**, 11658-11663 (2004).

16 Hermant, B. *et al.* Development of a one-step embryonic stem cell-based assay for the screening of sprouting angiogenesis. *BMC Biotechnol* **7**, 20 (2007).

17 Kivela, R. *et al.* VEGF-B-induced vascular growth leads to metabolic reprogramming and ischemia resistance in the heart. *EMBO Mol Med* **6**, 307-321 (2014).

18 Li, Y. *et al.* VEGF-B inhibits apoptosis via VEGFR-1-mediated suppression of the expression of BH3-only protein genes in mice and rats. *J Clin Invest* **118**, 913-923 (2008).

19 Lundin, L. *et al.* Selectively desulfated heparin inhibits fibroblast growth factor-induced mitogenicity and angiogenesis. *J Biol Chem* **275**, 24653-24660 (2000).

20 Mohan, R. *et al.* Curcuminoids inhibit the angiogenic response stimulated by fibroblast growth factor-2, including expression of matrix metalloproteinase gelatinase B. *J Biol Chem* **275**, 10405-10412 (2000).

21 Rybarczyk, B. J., Lawrence, S. O. & Simpson-Haidaris, P. J. Matrix-fibrinogen enhances wound closure by increasing both cell proliferation and migration. *Blood* **102**, 4035-4043 (2003).

22 Wimmer, R., Cseh, B., Maier, B., Scherrer, K. & Baccarini, M. Angiogenic sprouting requires the fine tuning of endothelial cell cohesion by the Raf-1/Rok-alpha complex. *Dev Cell* **22**, 158-171 (2012).

23 Zhang, F. *et al.* VEGF-B is dispensable for blood vessel growth but critical for their survival, and VEGF-B targeting inhibits pathological angiogenesis. *Proc Natl Acad Sci U S A* **106**, 6152-6157 (2009).

Figure. S1.


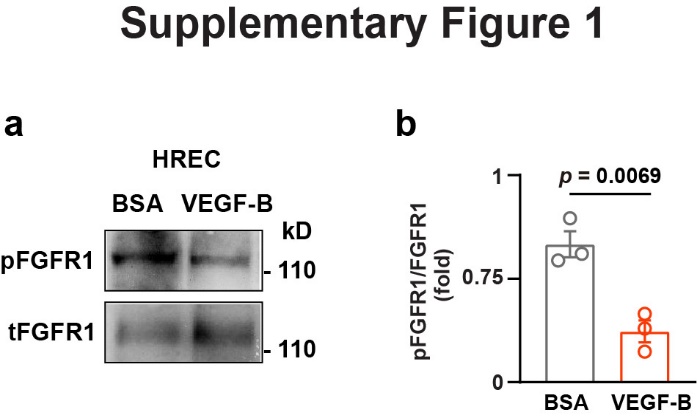


**Supplementary Figure 1. VEGF-B interacts with FGFR1.**

(**a,b**) Western blot showing that VEGF-B (50 ng/ml) treatment for 15 minutes reduced FGFR1 phosphorylation in HRECs. Two-tailed Student's *t*-test was used for data analysis. n = 3 each group. Data are mean ± s.e.m. The experiments were repeated three times.

Figure. S2.


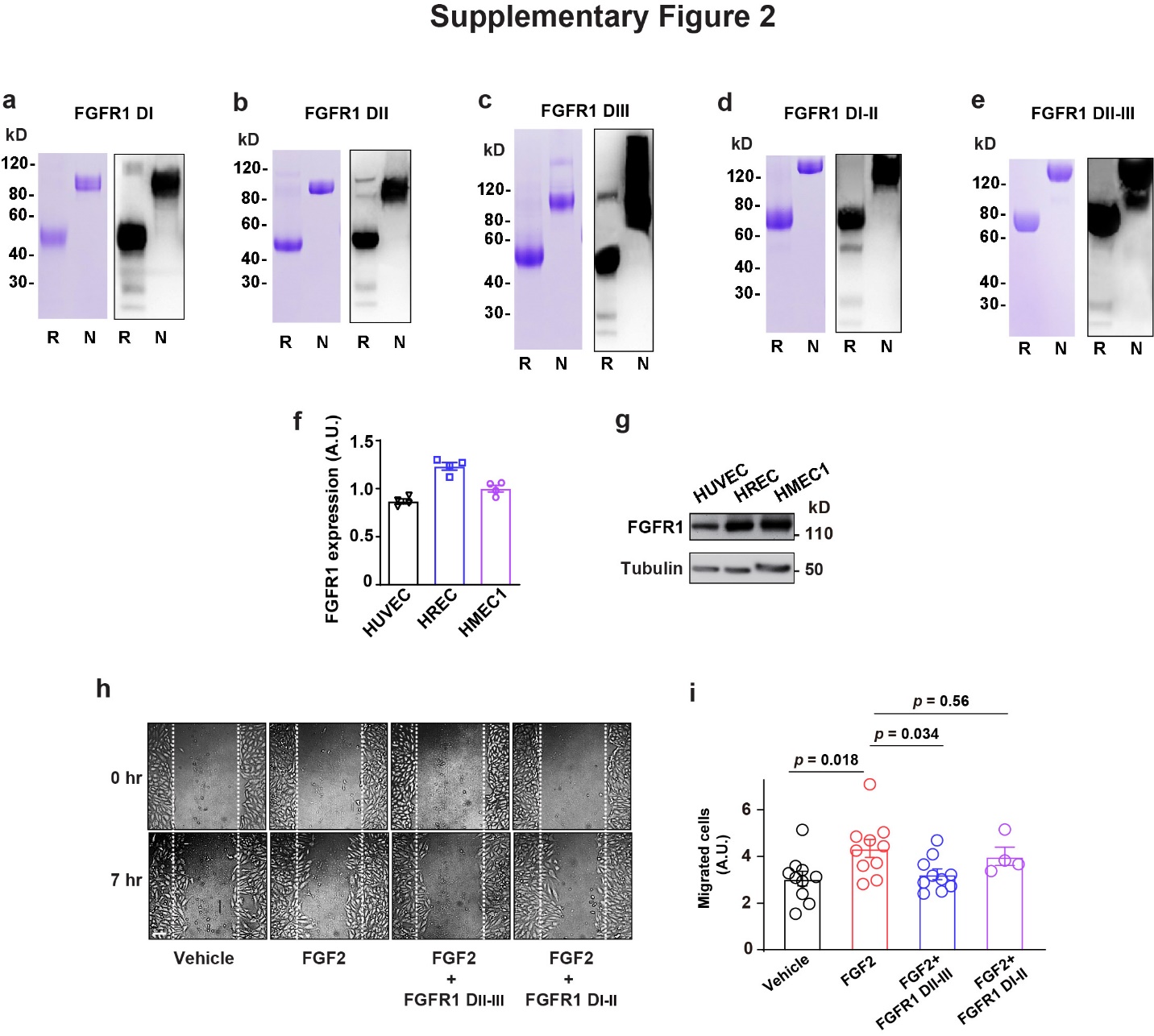


**Supplementary Figure 2. Different extracellular domains of FGFR1 display expected molecular weights**

(**a-e**) Coomassie blue staining and Western blots showing that the recombinant proteins of different extracellular domains (D) of FGFR1: FGFR1 DI (a), DII (b), DIII (c), DI-II (d) and DII-III (e) display expected molecular weights. R: reducing gel, N: non-reducing gel. (**f,g**) Real-time PCR (f) and Western blot (g) showing that FGFR1 is expressed in the three types of endothelial cells used in this study (HUVEC, HREC and HMEC1). (**h,i**) HREC migration assay showing that FGFR1 DII-III reduces FGF2 (50 ng/ml)-induced cell migration, while FGFR1 DI-II does not. Adjusted *p* values were from one-way ANOVA followed by Holm-Sidak post hoc analysis (number of comparisons, 3). n = 10, 10, 10 and 4 for vehicle, FGF2, FGF2+FGFR1 DII-III and FGF2+FGFR1 DI-II respectively. A.U.: arbitrary unit. Data are mean ± s.e.m. Scale bar: 50 µm, ns: *p* > 0.05. The experiments were repeated three times.

Figure. S3.


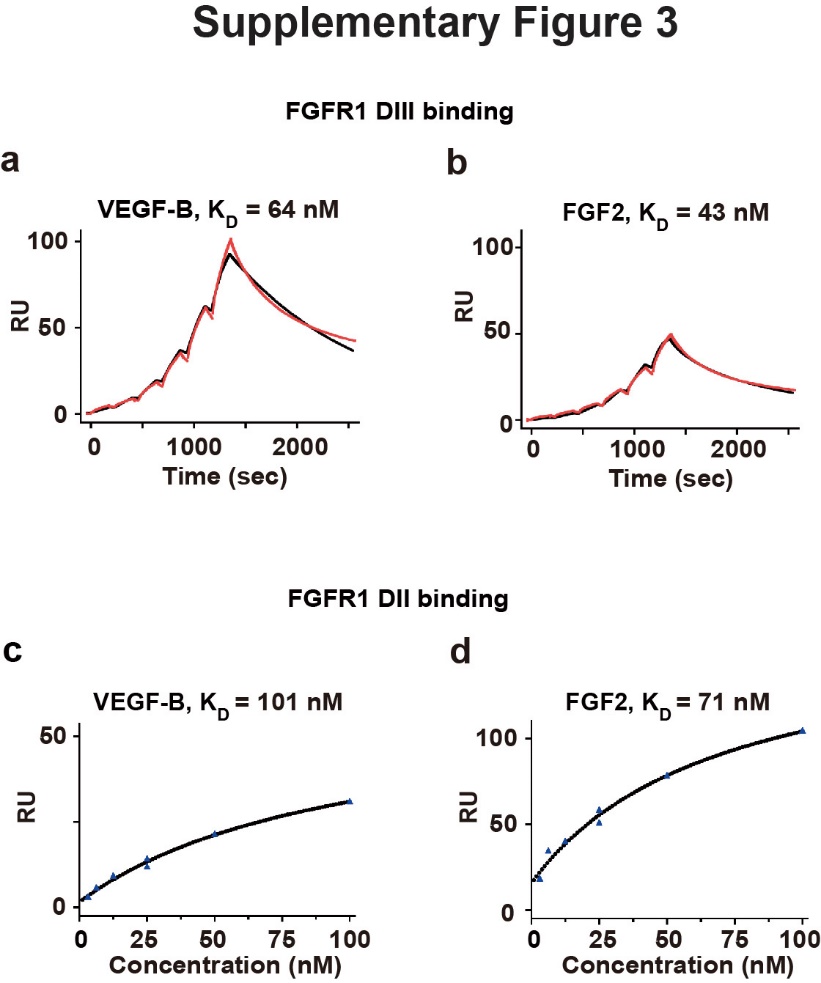


**Supplementary Figure 3. VEGF-B binds to FGFR1 DIII and FGFR1 DII with similar K_D_ values as FGF2.**

(**a,b**) Surface plasmon resonance assay showing that VEGF-B (a) binds to FGFR1 DIII with similar K_D_ values as FGF2 (b) (64 nM for VEGF-B and 43 nM for FGF2). The red lines are the RU values at different concentrations of FGFR1 DIII (12.5, 25, 50, 100, 200 and 400 nM). Black lines are the fitted curves. (**c,d**) Surface plasmon resonance assay showing that VEGF-B (c) binds to FGFR1 DII with similar K_D_ values as FGF2 (d) (101 nM for VEGF-B and 71 nM for FGF2). Colored crosses are the RU values at different concentrations of FGFR1 DII-Fc (3.125, 6.25, 12.5, 25, 50 and 100 nM). The black lines are the fitted curves.

Figure. S4.


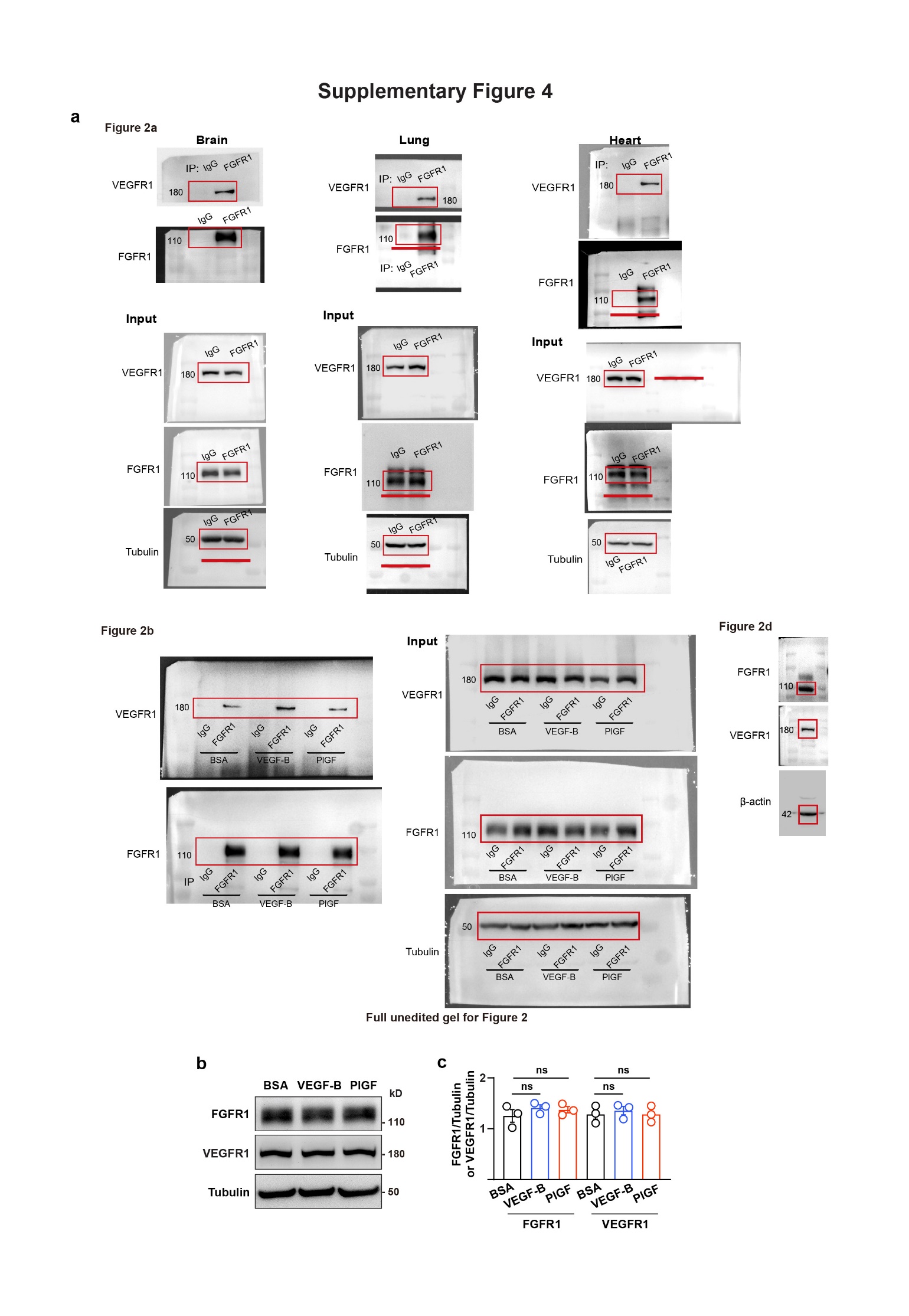


**Supplementary Figure 4. (a)** Full Western blot images for Fig. 2. **(b,c)** Western blots showing that FGFR1 and VEGFR1 levels were not changed after the treatment of VEGF-B or PlGF in the HRECs. n=3 each group. Data were analyzed using one-way ANOVA followed by Sidak post hoc analysis (number of comparisons, 2). The experiment was repeated three times.

Figure. S5.


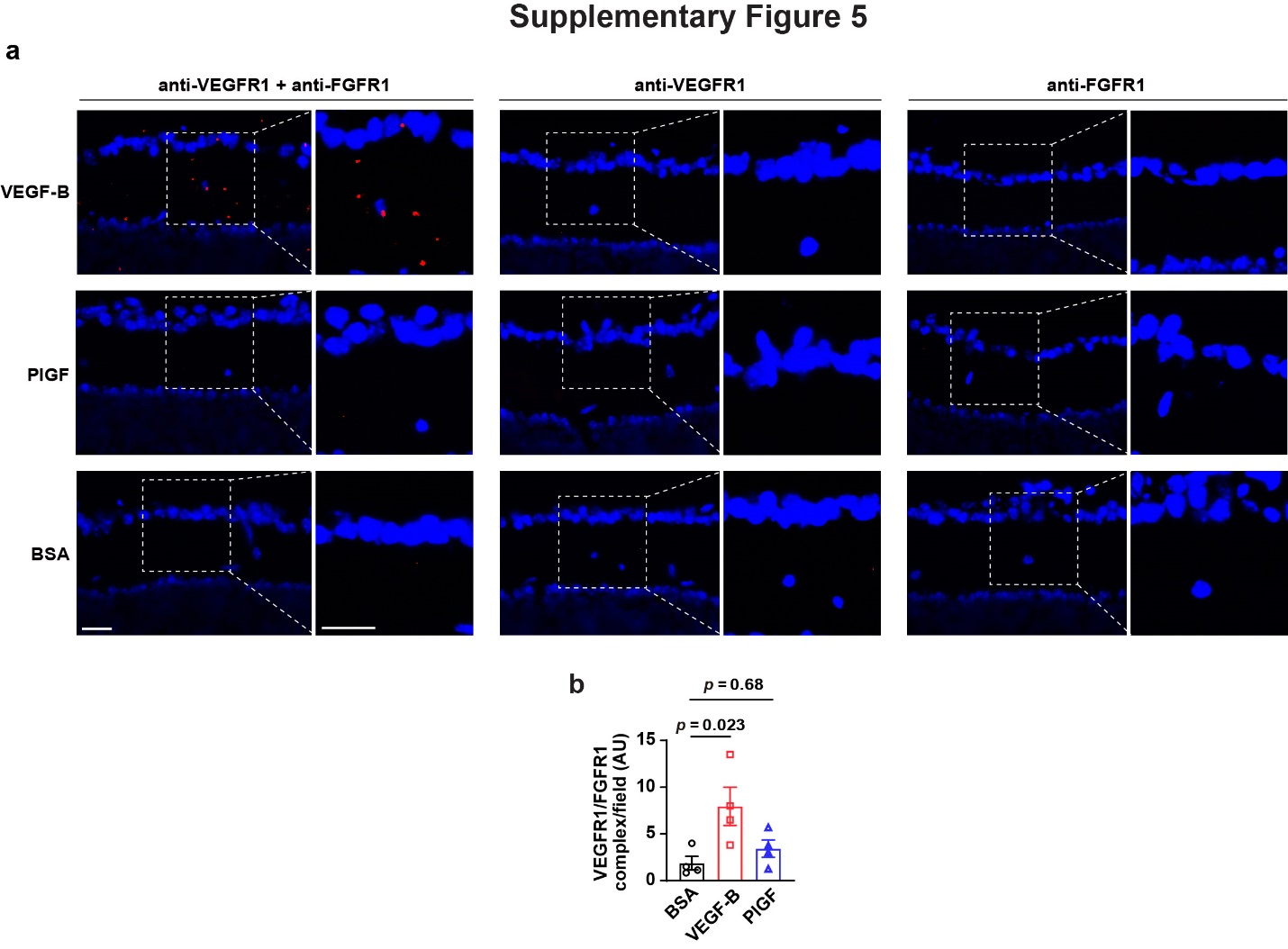


**Supplementary Figure 5. *In vivo* *in situ* proximity ligation assay of VEGFR1/FGFR1 complex in mouse retinae**

(**a,b**) Representative images (a) and quantifications (b) of *in situ* proximity ligation assays showing that VEGF-B (500 ng/eye), but not PlGF (500 ng/eye), induced VEGFR1/FGFR1 complex formation in mouse retinae *in vivo* 2 hours after intravitreal injection. Adding one antibody alone at a time (anti-FGFR1 or anti-VEGFR1) had no effect. Blue: DAPI, red: VEGFR1/FGFR1 complex. Data are mean ± s.e.m, n = 4 each group. Adjusted p values are from one-way ANOVA followed by Sidak post hoc analysis (number of comparisons, 2). Scale bars: 20 μm

Figure. S6.


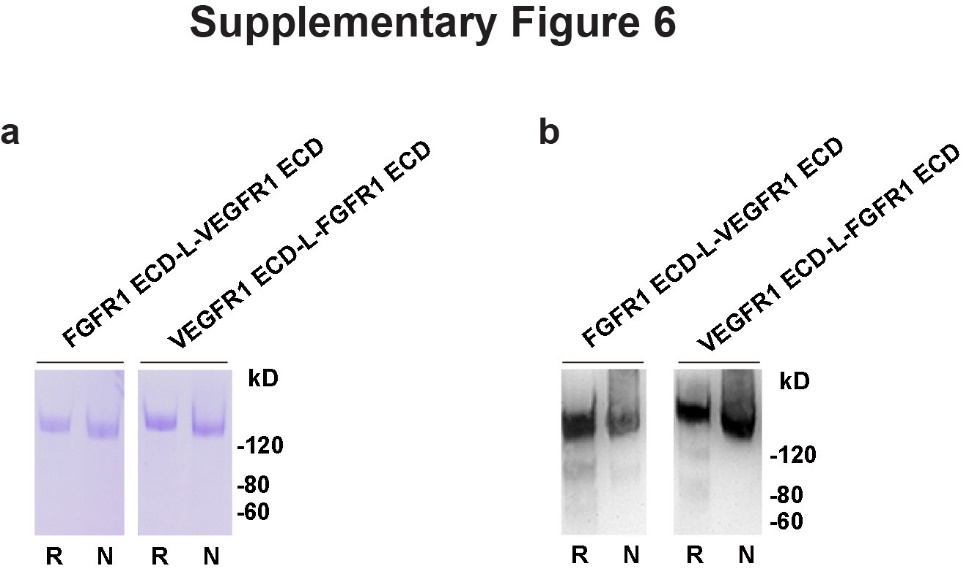


**Supplementary Figure 6. Recombinant proteins of FGFR1/VEGFR1 and VEGFR1/FGFR1 heterodimers display expected molecular weights.**

(**a,b**) Coomassie blue staining (a) and Western blots with anti-His antibody (b) under reducing (R) or non-reducing (N) conditions showing that the recombinant proteins of FGFR1/VEGFR1 and VEGFR1/FGFR1 heterodimers display expected molecular weights and do not form aggregates.

Figure. S7.


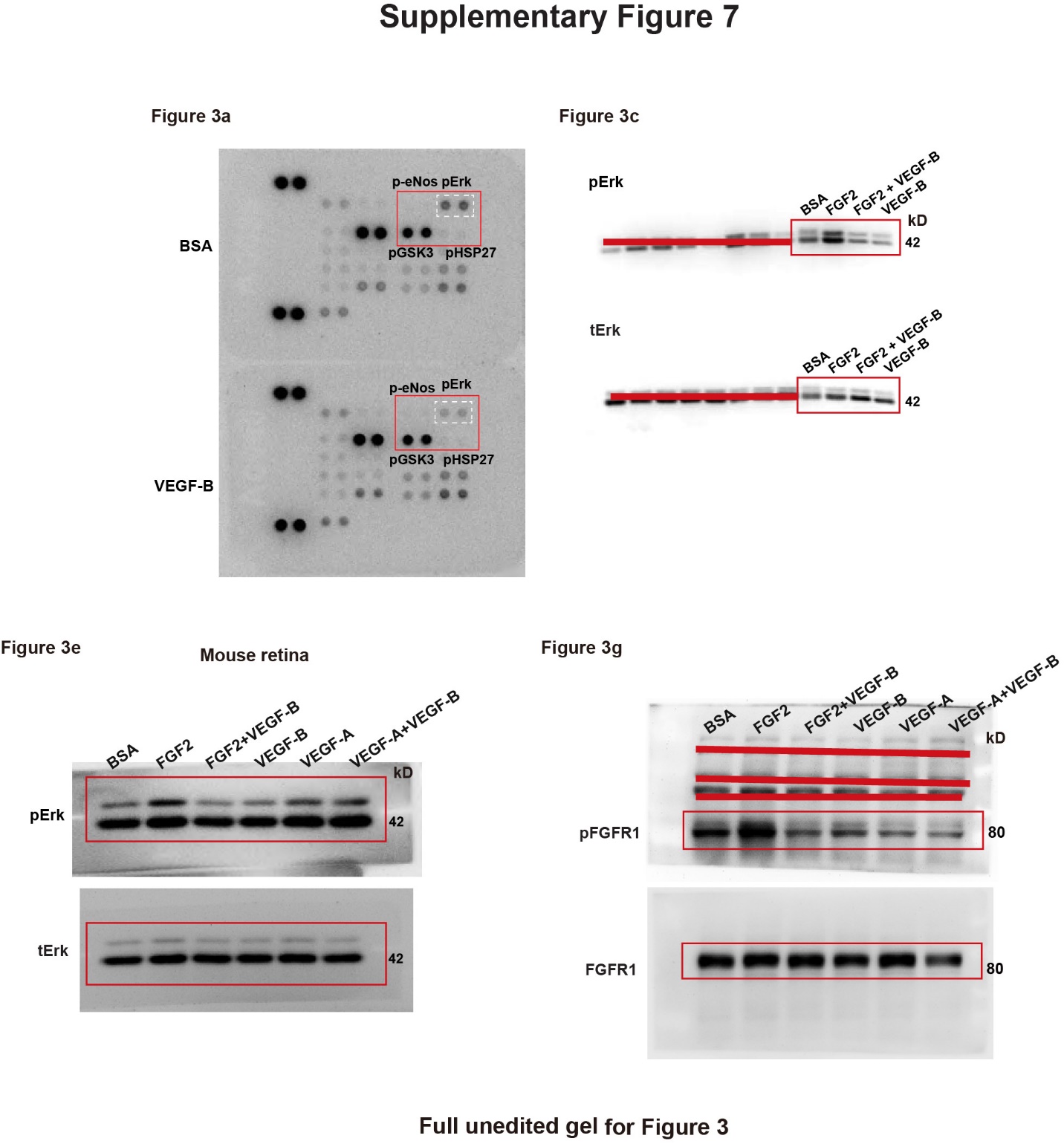


**Supplementary Figure 7. Full Western blot images for Fig. 3.**

Figure. S8.


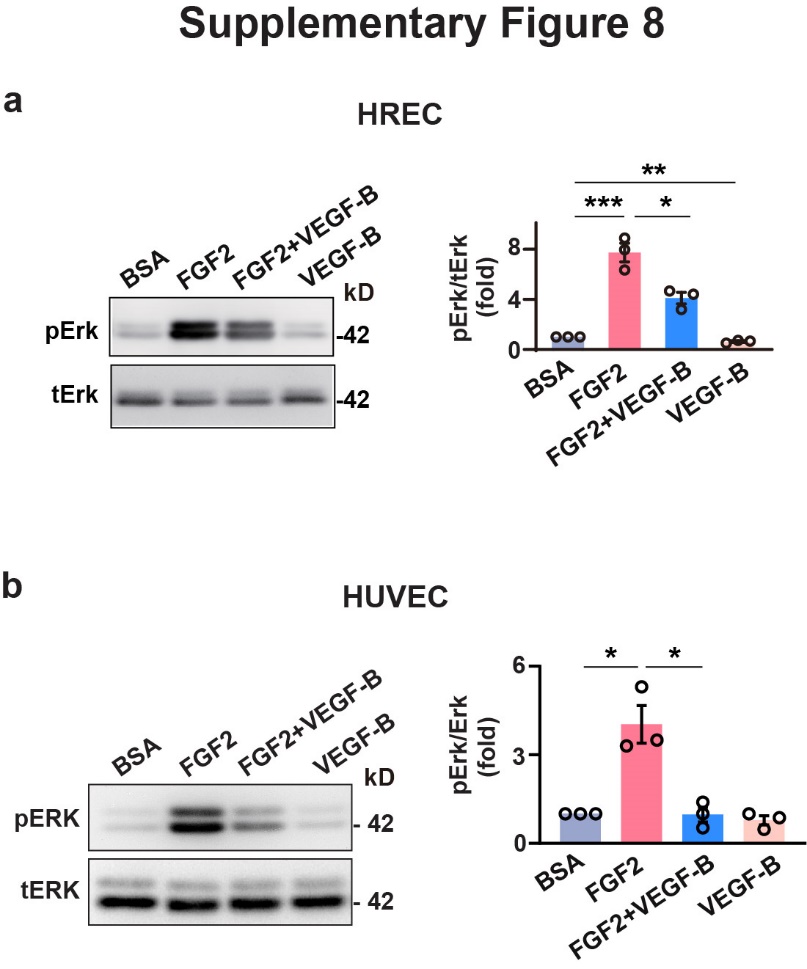


**Supplementary Figure 8. VEGF-B inhibits FGF2-induced Erk activation.**

(**a,b**) Western blots showing that VEGF-B inhibits FGF2-induced Erk activation in HRECs (a) and HUVECs (b). FGF2 (50 ng/ml), VEGF-B (100 ng/ml), FGF2 (50 ng/ml) + VEGF-B (100 ng/ml). The experiments were repeated three times.

Figure. S9.


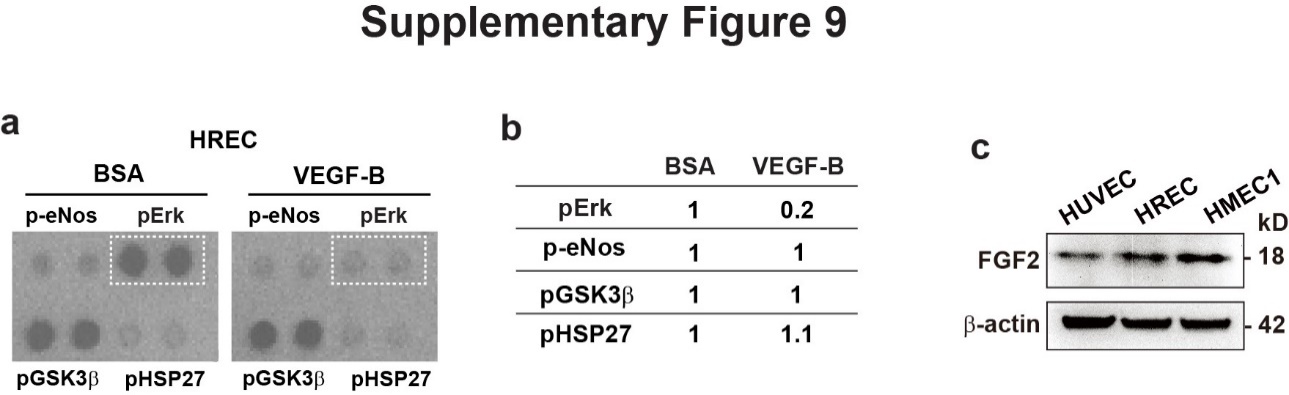


**Supplementary Figure 9. VEGF-B inhibits FGF2-induced Erk phosphorylation.**

(**a**) Images of phospho-MAPK antibody array screening assay showing that VEGF-B (50 ng/ml) inhibits Erk phosphorylation in HRECs. (**b**) Quantifications of the phospho-MAPK antibody array results in a. (**c**) Western blots showing that FGF2 is abundantly expressed in HUVECs, HRECs and HMEC1s.

Figure. S10.


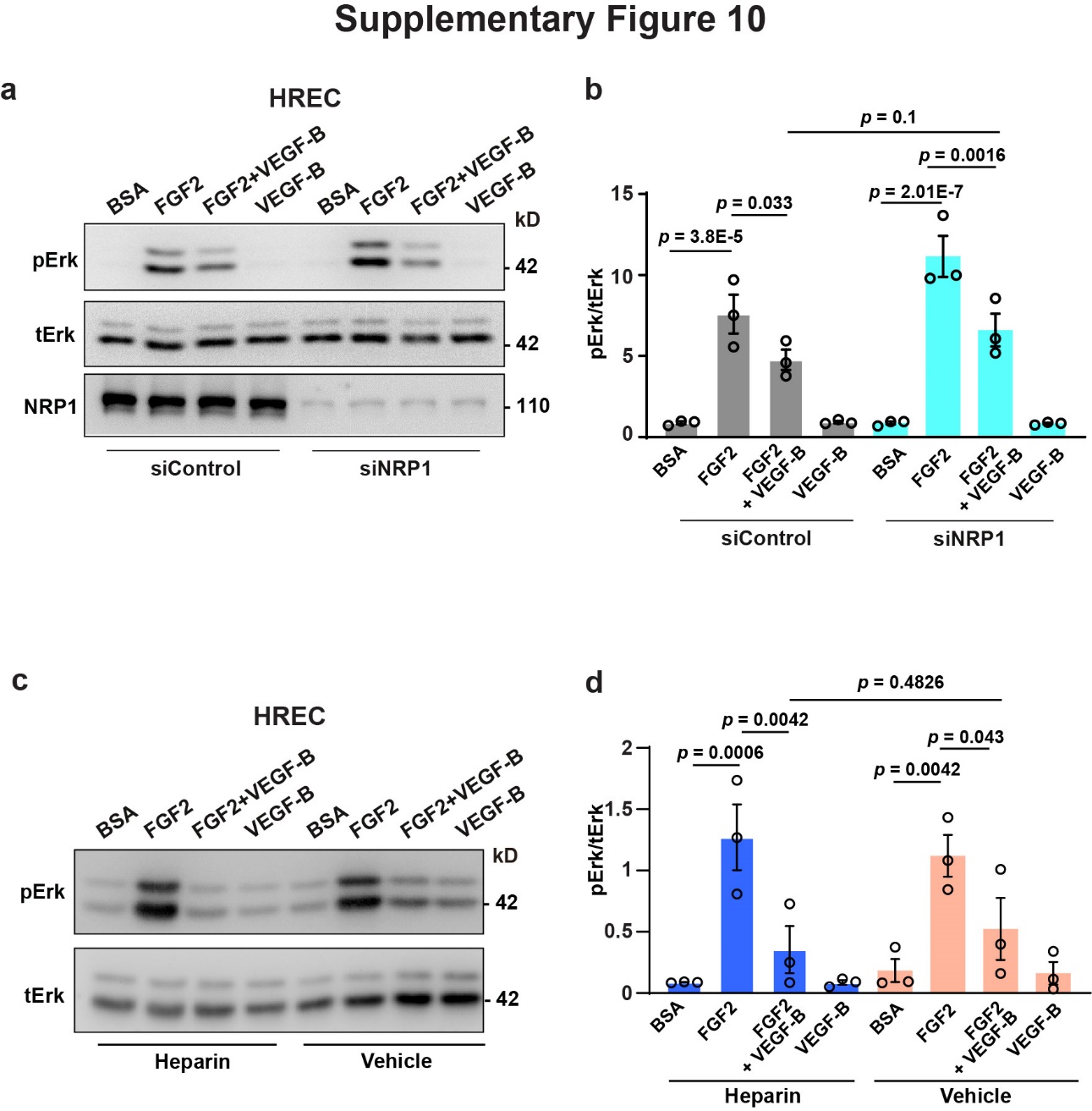


**Supplementary Figure 10.** **Effects of neuropilin 1 (NRP1) or heparin on the inhibitory effect of VEGF-B**

(**a,b**) Western blots showing that knockdown of NRP1 in HRECs did not affect the inhibitory effect of VEGF-B (100 ng/ml, 10 minutes treatment) on FGF2 (30 ng/ml)-induced Erk activation. One-way ANOVA followed by Holm-Sidak post hoc analysis was used (number of comparisons, 5). (**c,d**) Western blots showing that adding heparin to HRECs did not affect the inhibitory effect of VEGF-B (100 ng/ml, 10 minutes treatment) on FGF2 (30 ng/ml)-induced Erk activation. One-way ANOVA followed by Holm-Sidak post hoc analysis was used (number of comparisons, 5).

Figure. S11.


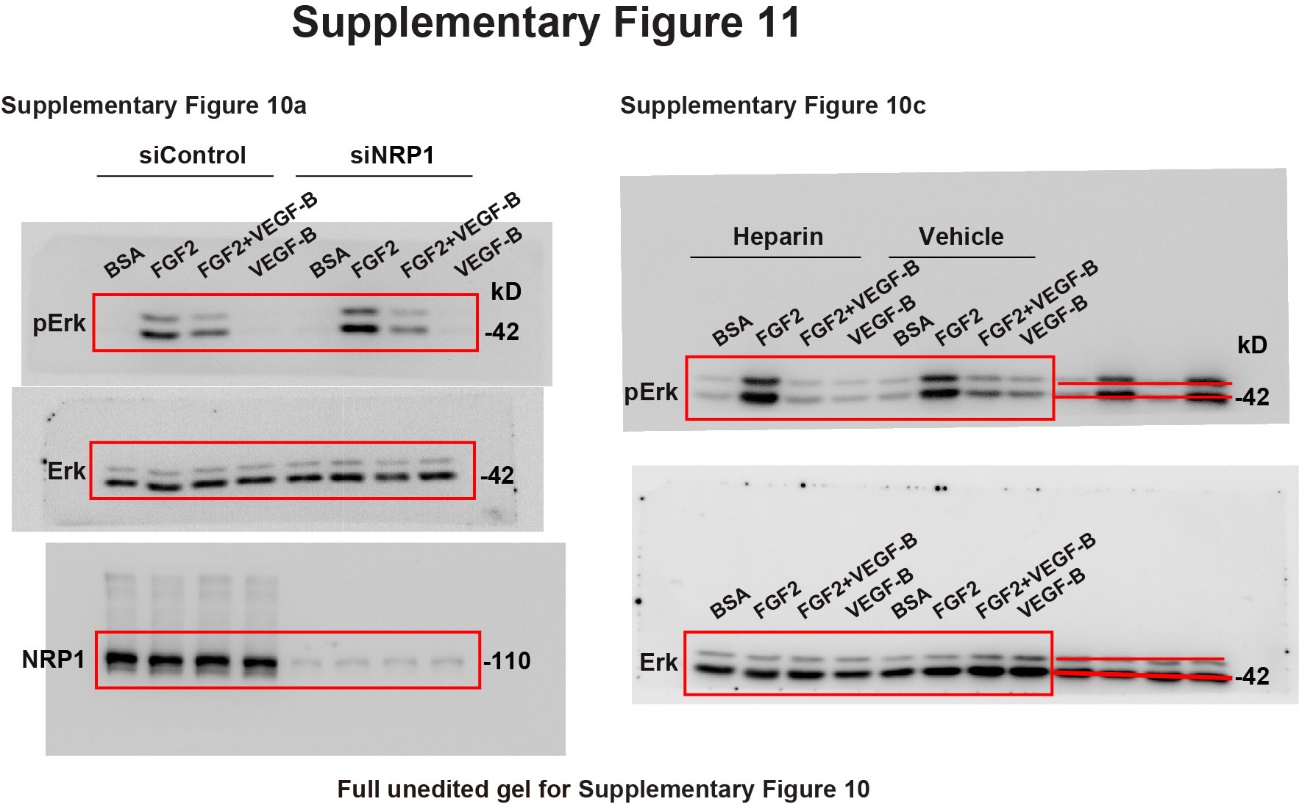


**Supplementary Figure 11. Full Western blot images for Supplementary Figure 10**

Figure. S12.


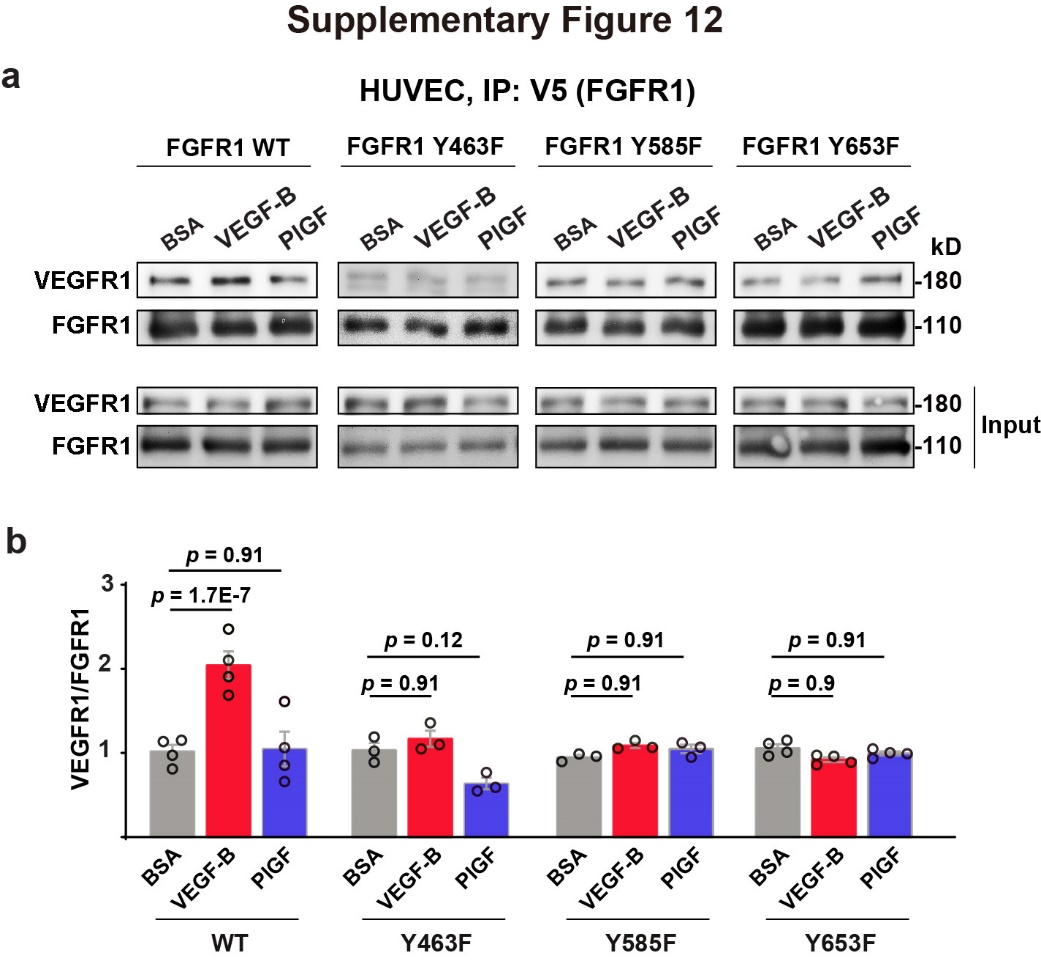


**Supplementary Figure 12. Effects of tyrosine residues Y463, Y585 and Y653 of FGFR1 on VEGF-B-induced VEGFR1/FGFR1 complex formation**

(**a**) Representative images of immunoprecipitation assays showing that VEGF-B induced complex formation of FGFR1/VEGFR1 in cells overexpressing wild-type FGFR1 (FGFR1 WT), but not in those overexpressing the FGFR1 mutants FGFR1 Y463F, FGFR1 Y585F or FGFR1 Y653F. (**b**) Quantifications of the bands in a. *P* values are from one-way ANOVA followed by Holm-Sidak post hoc analysis (number of comparisons, 8). n = 3 or 4

Figure. S13.


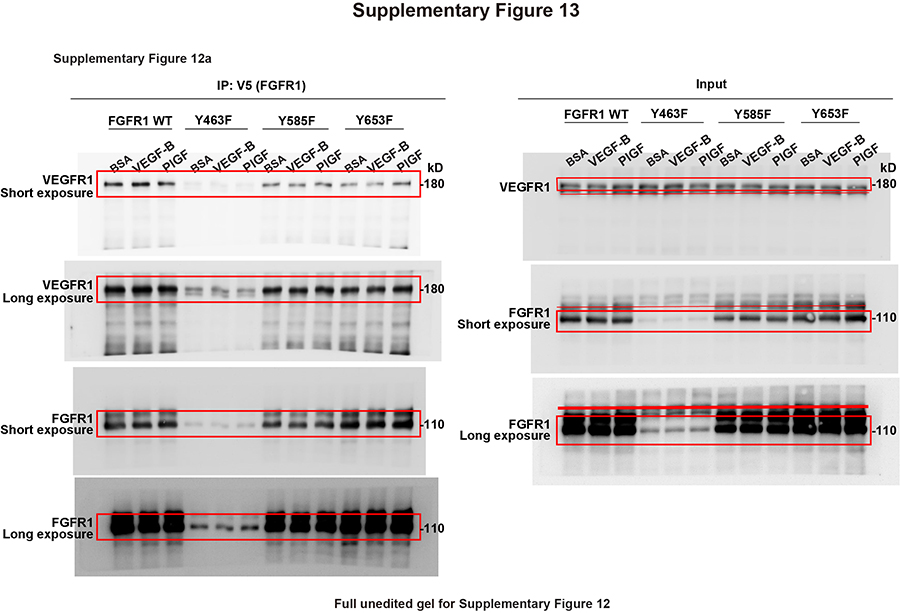


**Supplementary Figure 13. Full Western blot images for Supplementary Figure 12.**

Figure. S14.


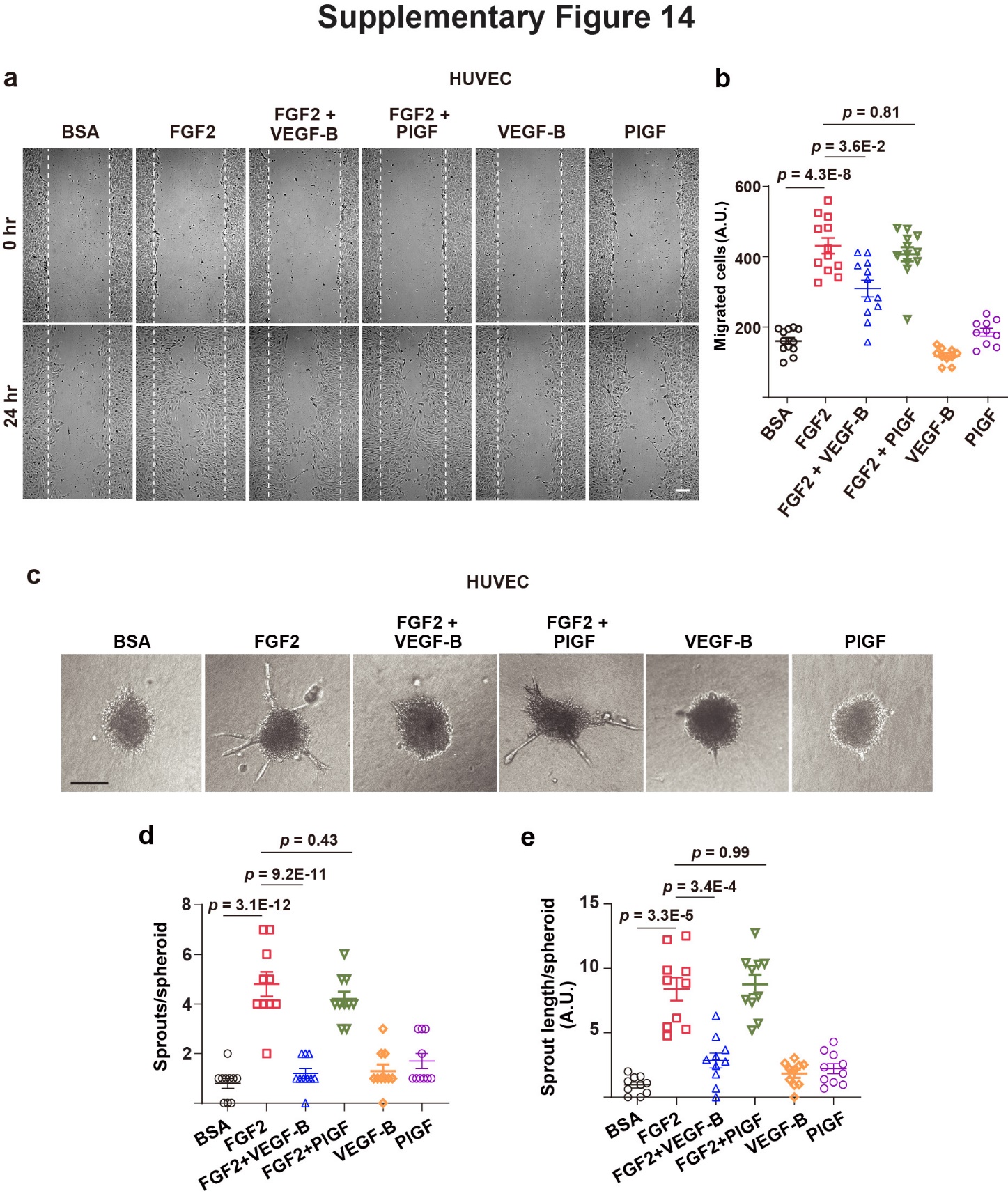


**Supplementary Figure 14. VEGF-B inhibits FGF2-induced HUVEC migration and spheroid formation.**

(**a,b**) Representative images (a) and corresponding quantifications (b) of monolayer HUVEC migration assay showing that VEGF-B, but not PlGF, inhibited FGF2-induced HUVEC migration. (n = 10-12 per group. The experiment was repeated three times.). Scale bar: 50 µm. (**c-e**) Representative images (c) of HUVEC spheroid sprouting assay and corresponding quantifications of the number (d) and length (e) of sprouts showing that VEGF-B (100 ng/ml), but not PlGF (100 ng/ml), inhibited FGF2 (50 ng/ml)-induced HUVEC spheroid sprouting (n = 10 per group. The experiment was repeated three times.). Scale bar for (c): 100 μm. For b and d, adjusted *p* values are from Welch ANOVA with Dunnett post hoc analysis (number of comparisons, 3). For e, one-way ANOVA followed by Sidak post hoc analysis (number of comparisons, 3) was used. A.U.: arbitrary unit. All data are mean ± s.e.m.

Figure. S15.


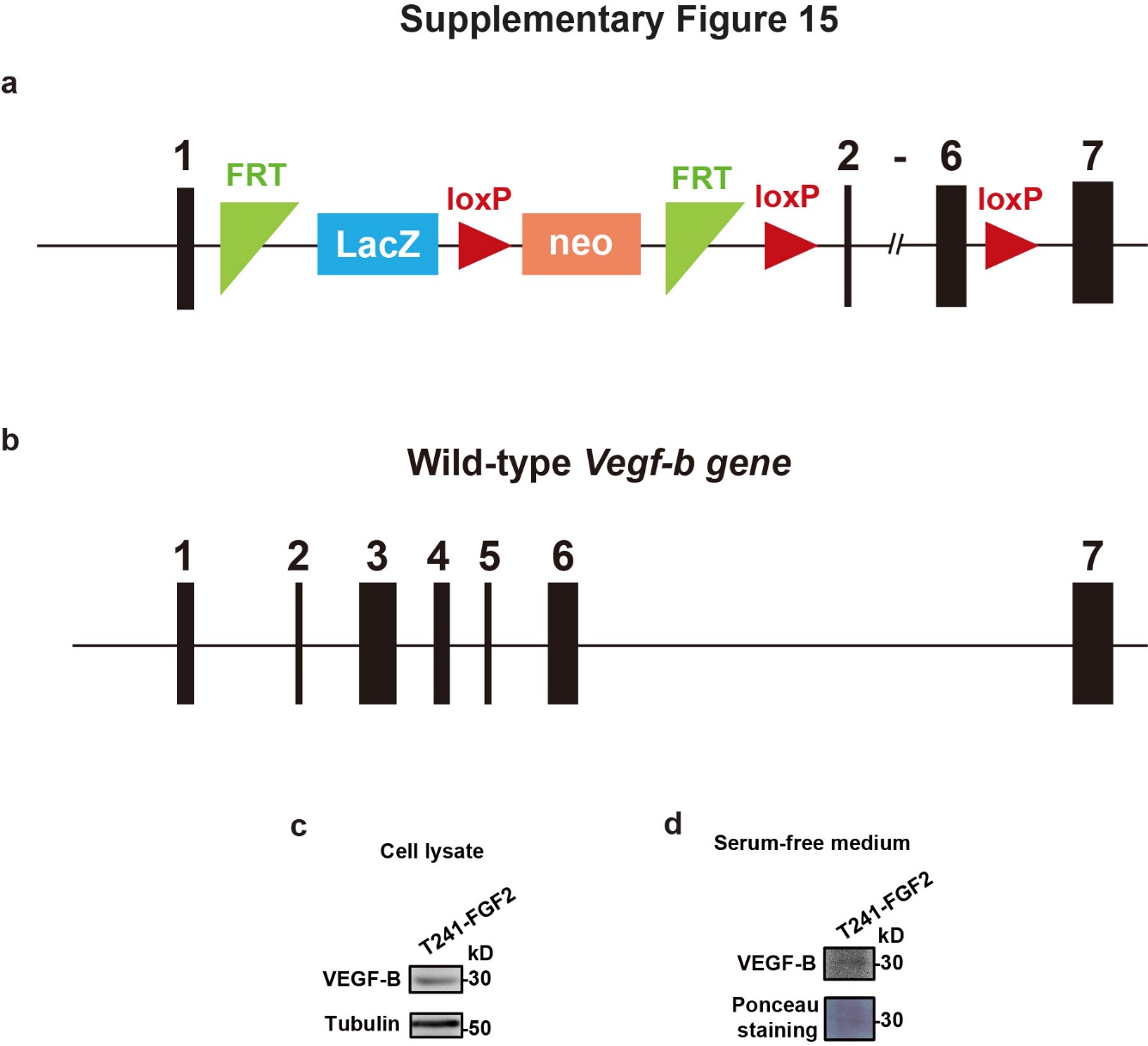


**Supplementary Figure 15. Schemes of the targeting vector and wild-type mouse *Vegf-b* gene.**

(**a**) Scheme showing the structure of the targeting vector for the deletion of mouse *Vegf-b* gene (Jackson Laboratory, <https://www.komp.org/>). An FRT site is followed by a lacZ cassette and a loxP site. The first loxP site is followed by a neomycin cassette, an FRT site and a loxP site. A third loxP site is inserted downstream of the targeted exons. (**b**) Scheme showing the structure of the wild-type mouse *Vegf-b* gene. Black boxes indicate the exons of *Vegf-b*. (**c,d**) Western blot detected VEGF-B expression in the cell lysate (c) and serum-free medium (d) of the T241-FGF2 cells.

Figure. S16.


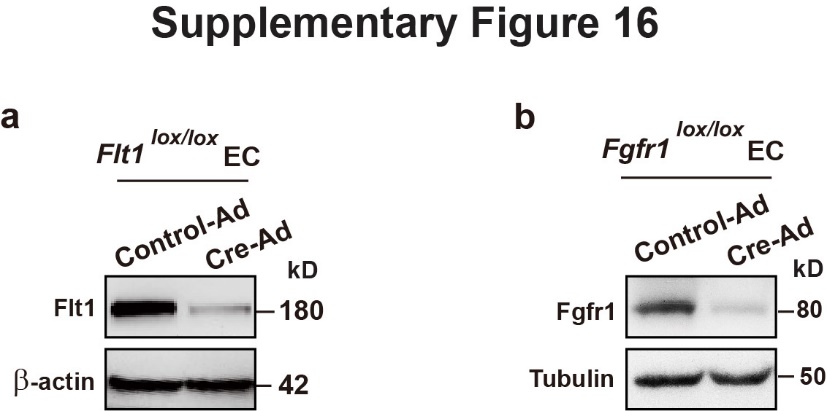


**Supplementary Figure 16. Deletion of *Flt1* or *Fgfr1* in primary mouse lung ECs and VEGFR1 activation in ECs**

(**a,b**) Western blots showing reduced expression of Flt1 (a) and Fgfr1 (b) in endothelial cells (ECs) isolated from lungs of *Flt1*^lox/lox^ or *Fgfr1*^lox/lox^ mice respectively upon treatment with adenovirus encoding the Cre enzyme (Cre-Ad) but not with control adenovirus (Control-Ad).

Figure. S17.

­­
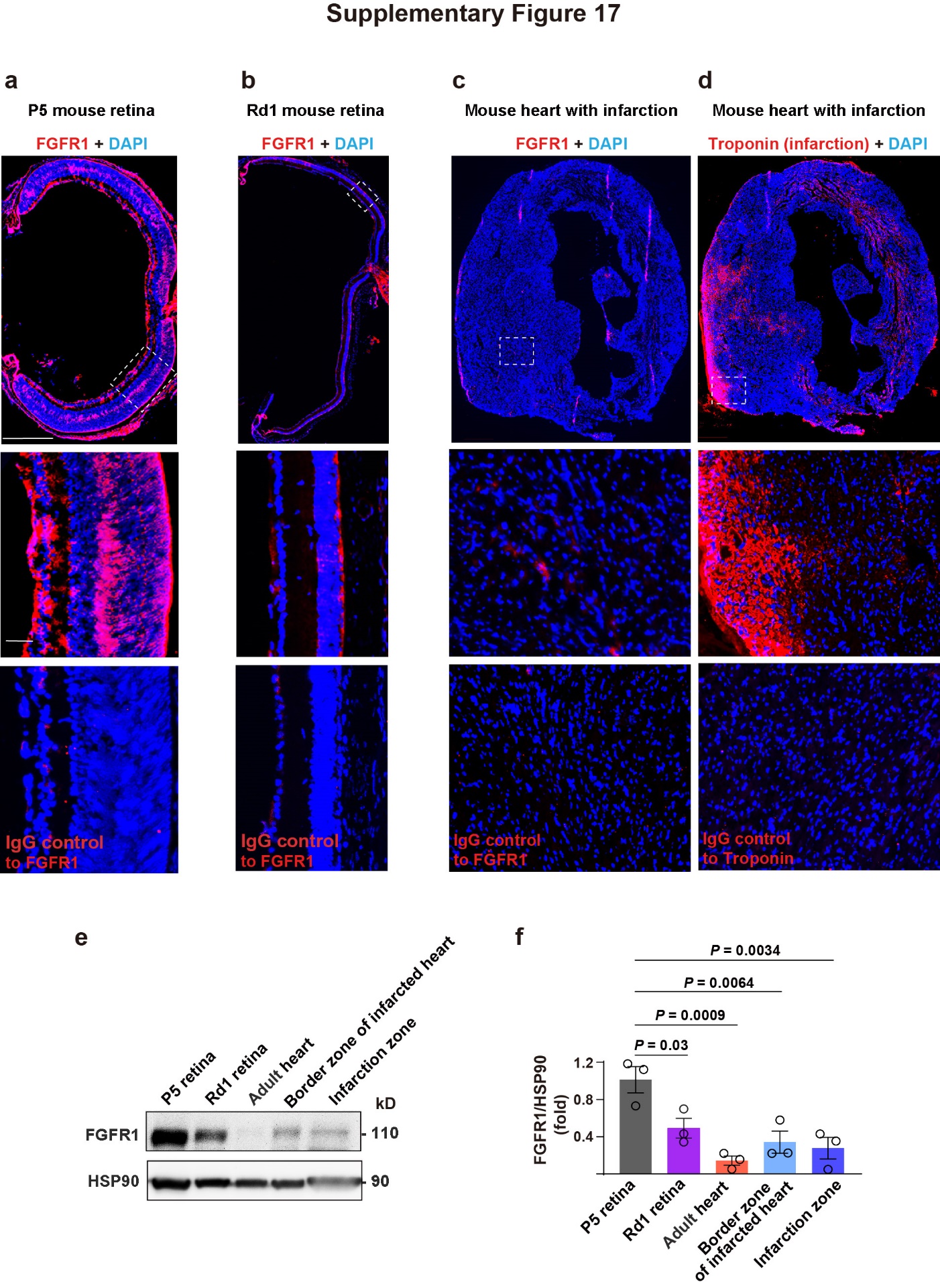


**Supplementary Figure 17. FGFR1 expression in different mouse tissues**

(**a-d**) Immunofluorescence staining showing that FGFR1 expression is high in P5 mouse retina (a) and low in degenerated rd1 retina (seven weeks old, b) and adult (two months old) mouse heart with infarction (c). Troponin staining indicates the infarction zones in the heart. Scale bars: 500 μm in the upper panels and 50 μm in the lower panels. IgG controls to FGFR1 or troponin showed no specific staining. (**e,f**) Western blots showing that FGFR1 expression level is high in P5 mouse retina and low degenerated rd1 retina (seven weeks old), adult (two months old) mouse heart, border zone of infarcted heart and infarction zone in the heart. Data are mean ± s.e.m. n = 3 each group. Adjusted *p* values are from one-way ANOVA followed by Sidak post hoc analysis (number of comparisons, 4). The experiment was repeated three times.

Table S1.

**Supplemental Table 1. VEGF-B is down-regulated in tumors. Over-expression of VEGF-B inhibits tumor growth and tumor angiogenesis. Low VEGF-B level is linked to poor survival of cancer patients.**

| **Cancer type** | **VEGF-B level and patient survival/tumor growth** | **PMID** | **Year** |
| --- | --- | --- | --- |
| Non–small cell lung cancer | Low VEGF-B expression is linked to poor patient survival. | 24145997 | 2014 |
| Non–small cell lung cancer | High VEGF-B expression is linked to better patient survival. | 19833826 | 2010 |
| Ileal carcinoids | Low VEGF-B expression is linked to poor patient survival. | 19816005 | 2009 |
| Breast cancer | VEGF-B is down-regulated in human breast cancer. | 19672262 | 2009 |
| Endometrial cancer | VEGF-B is down-regulated in human endometrial cancer. | 12942123 | 2003 |
| Myxoid/round-cell lipocarcinoma | VEGF-B is down-regulated in human myxoid/round-cell lipocarcinoma. | 20515481 | 2010 |
| Pancreatic neuroendocrine tumor | VEGF-B overexpression inhibits murine pancreatic neuroendocrine tumor. | 21124841 | 2010 |
| Melanoma | VEGF-B overexpression inhibits human melanoma in nude mice. | 25991856 | 2015 |
| Fibrosarcoma | VEGF-B overexpression inhibits murine fibrosarcoma. | 25991856 | 2015 |
| Breast cancer | Decreased VEGF-B levels in plasma of breast cancer patients | 30543720 | 2018 |
| Uveal melanoma | Decreased VEGF-B expression in uveal melanoma associated with higher tumor angiogenesis | 31337000 | 2019 |

Table S2.

**Supplementary Table 2. Primers used to produce the recombinant heterodimers of VEGFR1 and FGFR1 extracellular domains**

| **Heterodimers** | **Primers** | **Sequences** |
| --- | --- | --- |
| VEGFR1-L-FGFR1-L-His | Forward | CCAAGTTTAAACGGATCTCTAGCGAATTCCCGCCGCCACCATGGTCAGCTACTGGGACACCGGGGT |
|  | Reverse | GTCCATGTCCCTTCCCTCGATTCAGTGGTGGTGATGATGGTGCTCCAGGTAGAGCGGTGAGGTCAT |
| FGFR1-L-VEGFR1-L-His | Forward | CCAAGTTTAAACGGATCTCTAGCGAATTCCCGCCGCCACCATGTGGGGCTGGAAGTGCCTCCTCTTCT |
|  | Reverse | GTCCATGTCCCTTCCCTCGATTCAGTGGTGGTGATGATGGTGCTCCAGATTAGACTTGTCCGAGGTTCCTTG |

Table S3.

**Supplementary Table 3. Primers used for the site-directed mutagenesis of FGFR1**

| **FGFR1 mutants** | **Primers** | **Sequences** |
| --- | --- | --- |
| FGFR1 Y463F | Forward | GGCTGGAGTCTCCGAATTTGAGCTCCCTGAGGATCC |
|  | Reverse | GGATCCTCAGGGAGCTCAAATTCGGAGACTCCAGCC |
| FGFR1 Y583F | Forward | CCTCCTGGGCTGGAGTTCTGCTATAACCCCAGCCAC |
|  | Reverse | GTGGCTGGGGTTATAGCAGAACTCCAGCCCAGGAGG |
| FGFR1 Y585F | Forward | CCTGGGCTGGAGTACTGCTTTAACCCCAGCCACAACC |
|  | Reverse | GGTTGTGGCTGGGGTTAAAGCAGTACTCCAGCCCAGG |
| FGFR1 Y653F | Forward | ATTCATCATATCGACTTCTACAAGAAAACCACCAACGG |
|  | Reverse | CCGTTGGTGGTTTTCTTGTAGAAGTCGATATGATGAATG |
| FGFR1 Y654F | Forward | CATCCAATCGACTACTTCAAGAAAACCACCAACGGC |
|  | Reverse | GCCGTTGGTGGTTTTCTTGAAGTAGTCGATATGATG |
| FGFR1 Y766F | Forward | CCTCCAACCAGGAGTTTCTGGACCTGTCCATACC |
|  | Reverse | GGTATGGACAGGTCCAGAAACTCCTGGTTGGAGG |

Table S4.

**Supplementary Table 4.**

| **Coding sequences for the VEGFR1-L-FGFR1-His recombinant protein** |
| --- |

ATGGTCAGCTACTGGGACACCGGGGTCCTGCTGTGCGCGCTGCTCAGCTGTCTGCTTCTCACAGGATCTAGTTCAGGTTCAAAATTAAAAGATCCTGAACTGAGTTTAAAAGGCACCCAGCACATCATGCAAGCAGGCCAGACACTGCATCTCCAATGCAGGGGGGAAGCAGCCCATAAATGGTCTTTGCCTGAAATGGTGAGTAAGGAAAGCGAAAGGCTGAGCATAACTAAATCTGCCTGTGGAAGAAATGGCAAACAATTCTGCAGTACTTTAACCTTGAACACAGCTCAAGCAAACCACACTGGCTTCTACAGCTGCAAATATCTAGCTGTACCTACTTCAAAGAAGAAGGAAACAGAATCTGCAATCTATATATTTATTAGTGATACAGGTAGACCTTTCGTAGAGATGTACAGTGAAATCCCCGAAATTATACACATGACTGAAGGAAGGGAGCTCGTCATTCCCTGCCGGGTTACGTCACCTAACATCACTGTTACTTTAAAAAAGTTTCCACTTGACACTTTGATCCCTGATGGAAAACGCATAATCTGGGACAGTAGAAAGGGCTTCATCATATCAAATGCAACGTACAAAGAAATAGGGCTTCTGACCTGTGAAGCAACAGTCAATGGGCATTTGTATAAGACAAACTATCTCACACATCGACAAACCAATACAATCATAGATGTCCAAATAAGCACACCACGCCCAGTCAAATTACTTAGAGGCCATACTCTTGTCCTCAATTGTACTGCTACCACTCCCTTGAACACGAGAGTTCAAATGACCTGGAGTTACCCTGATGAAAAAAATAAGAGAGCTTCCGTAAGGCGACGAATTGACCAAAGCAATTCCCATGCCAACATATTCTACAGTGTTCTTACTATTGACAAAATGCAGAACAAAGACAAAGGACTTTATACTTGTCGTGTAAGGAGTGGACCATCATTCAAATCTGTTAACACCTCAGTGCATATATATGATAAAGCATTCATCACTGTGAAACATCGAAAACAGCAGGTGCTTGAAACCGTAGCTGGCAAGCGGTCTTACCGGCTCTCTATGAAAGTGAAGGCATTTCCCTCGCCGGAAGTTGTATGGTTAAAAGATGGGTTACCTGCGACTGAGAAATCTGCTCGCTATTTGACTCGTGGCTACTCGTTAATTATCAAGGACGTAACTGAAGAGGATGCAGGGAATTATACAATCTTGCTGAGCATAAAACAGTCAAATGTGTTTAAAAACCTCACTGCCACTCTAATTGTCAATGTGAAACCCCAGATTTACGAAAAGGCCGTGTCATCGTTTCCAGACCCGGCTCTCTACCCACTGGGCAGCAGACAAATCCTGACTTGTACCGCATATGGTATCCCTCAACCTACAATCAAGTGGTTCTGGCACCCCTGTAACCATAATCATTCCGAAGCAAGGTGTGACTTTTGTTCCAATAATGAAGAGTCCTTTATCCTGGATGCTGACAGCAACATGGGAAACAGAATTGAGAGCATCACTCAGCGCATGGCAATAATAGAAGGAAAGAATAAGATGGCTAGCACCTTGGTTGTGGCTGACTCTAGAATTTCTGGAATCTACATTTGCATAGCTTCCAATAAAGTTGGGACTGTGGGAAGAAACATAAGCTTTTATATCACAGATGTGCCAAATGGGTTTCATGTTAACTTGGAAAAAATGCCGACGGAAGGAGAGGACCTGAAACTGTCTTGCACAGTTAACAAGTTCTTATACAGAGACGTTACTTGGATTTTACTGCGGACAGTTAATAACAGAACAATGCACTACAGTATTAGCAAGCAAAAAATGGCCATCACTAAGGAGCACTCCATCACTCTTAATCTTACCATCATGAATGTTTCCCTGCAAGATTCAGGCACCTATGCCTGCAGAGCCAGGAATGTATACACAGGGGAAGAAATCCTCCAGAAGAAAGAAATTACAATCAGAGATCAGGAAGCACCATACCTCCTGCGAAACCTCAGTGATCACACAGTGGCCATCAGCAGTTCCACCACTTTAGACTGTCATGCTAATGGTGTCCCCGAGCCTCAGATCACTTGGTTTAAAAACAACCACAAAATACAACAAGAGCCTGGAATTATTTTAGGACCAGGAAGCAGCACGCTGTTTATTGAAAGAGTCACAGAAGAGGATGAAGGTGTCTATCACTGCAAAGCCACCAACCAGAAGGGCTCTGTGGAAAGTTCAGCATACCTCACTGTTCAAGGAACCTCGGACAAGTCTAATCTGGAGGGAGGAGGAGGAAGCGGAGGAGGAGGATCTGGAGGAGGAGGATCTAGGCCAGCCCCAACCTTGCCCGAACAAGCTCAGCCCTGGGGAGTCCCTGTGGAAGTGGAGTCTCTCCTGGTCCACCCTGGCGACCTGCTACAGCTTCGCTGTCGGCTTCGCGATGATGTGCAGAGCATCAACTGGCTGCGGGATGGGGTGCAGCTGGTGGAGAGCAACCGTACCCGCATCACAGGGGAGGAGGTGGAGGTGCGGGACTCCATCCCCGCTGACTCTGGCCTCTACGCTTGCGTGACCAGCAGCCCCTCTGGCAGCGATACCACCTACTTCTCCGTCAATGTCTCAGATGCACTCCCATCCTCGGAAGATGATGACGACGACGATGACTCCTCCTCGGAGGAGAAAGAGACGGACAACACCAAACCAAACCCTGTAGCTCCCTACTGGACATCCCCAGAGAAAATGGAGAAGAAACTGCATGCGGTGCCCGCTGCCAAGACGGTGAAGTTCAAGTGCCCGTCGAGTGGGACACCCAACCCCACTCTGCGCTGGTTGAAAAATGGCAAAGAGTTTAAGCCTGACCACCGAATTGGAGGCTACAAGGTTCGCTATGCCACCTGGAGCATCATAATGGATTCTGTGGTGCCTTCTGACAAGGGCAACTACACCTGCATCGTGGAGAATGAGTATGGGAGCATCAACCACACCTACCAGCTTGACGTCGTGGAACGATCTCCGCACCGACCCATCCTTCAGGCAGGGCTGCCTGCCAACAAGACAGTGGCCCTGGGCAGCAATGTGGAGTTCATGTGTAAGGTGTACAGCGATCCGCAGCCTCACATTCAGTGGCTGAAGCACATCGAGGTGAACGGGAGTAAGATCGGGCCAGACAACTTGCCGTATGTCCAGATCCTGAAGACTGCTGGAGTTAATACCACCGACAAGGAAATGGAGGTGCTTCATCTACGGAATGTCTCCTTTGAGGATGCGGGGGAGTATACGTGCTTGGCGGGTAACTCTATCGGACTCTCCCATCACTCTGCATGGTTGACCGTTCTGGAAGCCCTGGAAGAGAGACCAGCTGTGATGACCTCACCGCTCTACCTGGAG**CACCATCATCACCACCAC**TGA

|  |
| --- |

Underlined: linker; Bold: His tag.

Table S5.

**Supplementary Table 4.**

| **Coding sequences for the FGFR1-L-VEGFR1-His recombinant protein** |
| --- |

ATGTGGGGCTGGAAGTGCCTCCTCTTCTGGGCTGTGCTGGTCACAGCCACTCTCTGCACTGCCAGGCCAGCCCCAACCTTGCCCGAACAAGCTCAGCCCTGGGGAGTCCCTGTGGAAGTGGAGTCTCTCCTGGTCCACCCTGGCGACCTGCTACAGCTTCGCTGTCGGCTTCGCGATGATGTGCAGAGCATCAACTGGCTGCGGGATGGGGTGCAGCTGGTGGAGAGCAACCGTACCCGCATCACAGGGGAGGAGGTGGAGGTGCGGGACTCCATCCCCGCTGACTCTGGCCTCTACGCTTGCGTGACCAGCAGCCCCTCTGGCAGCGATACCACCTACTTCTCCGTCAATGTCTCAGATGCACTCCCATCCTCGGAAGATGATGACGACGACGATGACTCCTCCTCGGAGGAGAAAGAGACGGACAACACCAAACCAAACCCTGTAGCTCCCTACTGGACATCCCCAGAGAAAATGGAGAAGAAACTGCATGCGGTGCCCGCTGCCAAGACGGTGAAGTTCAAGTGCCCGTCGAGTGGGACACCCAACCCCACTCTGCGCTGGTTGAAAAATGGCAAAGAGTTTAAGCCTGACCACCGAATTGGAGGCTACAAGGTTCGCTATGCCACCTGGAGCATCATAATGGATTCTGTGGTGCCTTCTGACAAGGGCAACTACACCTGCATCGTGGAGAATGAGTATGGGAGCATCAACCACACCTACCAGCTTGACGTCGTGGAACGATCTCCGCACCGACCCATCCTTCAGGCAGGGCTGCCTGCCAACAAGACAGTGGCCCTGGGCAGCAATGTGGAGTTCATGTGTAAGGTGTACAGCGATCCGCAGCCTCACATTCAGTGGCTGAAGCACATCGAGGTGAACGGGAGTAAGATCGGGCCAGACAACTTGCCGTATGTCCAGATCCTGAAGACTGCTGGAGTTAATACCACCGACAAGGAAATGGAGGTGCTTCATCTACGGAATGTCTCCTTTGAGGATGCGGGGGAGTATACGTGCTTGGCGGGTAACTCTATCGGACTCTCCCATCACTCTGCATGGTTGACCGTTCTGGAAGCCCTGGAAGAGAGACCAGCTGTGATGACCTCACCGCTCTACCTGGAGGGAGGAGGAGGAAGCGGAGGAGGAGGATCTGGAGGAGGAGGATCTTCAAAATTAAAAGATCCTGAACTGAGTTTAAAAGGCACCCAGCACATCATGCAAGCAGGCCAGACACTGCATCTCCAATGCAGGGGGGAAGCAGCCCATAAATGGTCTTTGCCTGAAATGGTGAGTAAGGAAAGCGAAAGGCTGAGCATAACTAAATCTGCCTGTGGAAGAAATGGCAAACAATTCTGCAGTACTTTAACCTTGAACACAGCTCAAGCAAACCACACTGGCTTCTACAGCTGCAAATATCTAGCTGTACCTACTTCAAAGAAGAAGGAAACAGAATCTGCAATCTATATATTTATTAGTGATACAGGTAGACCTTTCGTAGAGATGTACAGTGAAATCCCCGAAATTATACACATGACTGAAGGAAGGGAGCTCGTCATTCCCTGCCGGGTTACGTCACCTAACATCACTGTTACTTTAAAAAAGTTTCCACTTGACACTTTGATCCCTGATGGAAAACGCATAATCTGGGACAGTAGAAAGGGCTTCATCATATCAAATGCAACGTACAAAGAAATAGGGCTTCTGACCTGTGAAGCAACAGTCAATGGGCATTTGTATAAGACAAACTATCTCACACATCGACAAACCAATACAATCATAGATGTCCAAATAAGCACACCACGCCCAGTCAAATTACTTAGAGGCCATACTCTTGTCCTCAATTGTACTGCTACCACTCCCTTGAACACGAGAGTTCAAATGACCTGGAGTTACCCTGATGAAAAAAATAAGAGAGCTTCCGTAAGGCGACGAATTGACCAAAGCAATTCCCATGCCAACATATTCTACAGTGTTCTTACTATTGACAAAATGCAGAACAAAGACAAAGGACTTTATACTTGTCGTGTAAGGAGTGGACCATCATTCAAATCTGTTAACACCTCAGTGCATATATATGATAAAGCATTCATCACTGTGAAACATCGAAAACAGCAGGTGCTTGAAACCGTAGCTGGCAAGCGGTCTTACCGGCTCTCTATGAAAGTGAAGGCATTTCCCTCGCCGGAAGTTGTATGGTTAAAAGATGGGTTACCTGCGACTGAGAAATCTGCTCGCTATTTGACTCGTGGCTACTCGTTAATTATCAAGGACGTAACTGAAGAGGATGCAGGGAATTATACAATCTTGCTGAGCATAAAACAGTCAAATGTGTTTAAAAACCTCACTGCCACTCTAATTGTCAATGTGAAACCCCAGATTTACGAAAAGGCCGTGTCATCGTTTCCAGACCCGGCTCTCTACCCACTGGGCAGCAGACAAATCCTGACTTGTACCGCATATGGTATCCCTCAACCTACAATCAAGTGGTTCTGGCACCCCTGTAACCATAATCATTCCGAAGCAAGGTGTGACTTTTGTTCCAATAATGAAGAGTCCTTTATCCTGGATGCTGACAGCAACATGGGAAACAGAATTGAGAGCATCACTCAGCGCATGGCAATAATAGAAGGAAAGAATAAGATGGCTAGCACCTTGGTTGTGGCTGACTCTAGAATTTCTGGAATCTACATTTGCATAGCTTCCAATAAAGTTGGGACTGTGGGAAGAAACATAAGCTTTTATATCACAGATGTGCCAAATGGGTTTCATGTTAACTTGGAAAAAATGCCGACGGAAGGAGAGGACCTGAAACTGTCTTGCACAGTTAACAAGTTCTTATACAGAGACGTTACTTGGATTTTACTGCGGACAGTTAATAACAGAACAATGCACTACAGTATTAGCAAGCAAAAAATGGCCATCACTAAGGAGCACTCCATCACTCTTAATCTTACCATCATGAATGTTTCCCTGCAAGATTCAGGCACCTATGCCTGCAGAGCCAGGAATGTATACACAGGGGAAGAAATCCTCCAGAAGAAAGAAATTACAATCAGAGATCAGGAAGCACCATACCTCCTGCGAAACCTCAGTGATCACACAGTGGCCATCAGCAGTTCCACCACTTTAGACTGTCATGCTAATGGTGTCCCCGAGCCTCAGATCACTTGGTTTAAAAACAACCACAAAATACAACAAGAGCCTGGAATTATTTTAGGACCAGGAAGCAGCACGCTGTTTATTGAAAGAGTCACAGAAGAGGATGAAGGTGTCTATCACTGCAAAGCCACCAACCAGAAGGGCTCTGTGGAAAGTTCAGCATACCTCACTGTTCAAGGAACCTCGGACAAGTCTAATCTGGAG**CACCATCATCACCACCAC**TGA

|  |
| --- |

Underlined: linker; Bold: His tag.
